# Anaphase B

**DOI:** 10.3390/biology5040051

**Published:** 2016-12-08

**Authors:** Jonathan M. Scholey, Gul Civelekoglu-Scholey, Ingrid Brust-Mascher

**Affiliations:** 1 Department of Molecular and Cell Biology, University of California, Davis, CA 95616, USA; egcivelekogluscholey@ucdavis.edu; 2 Department of Anatomy, Physiology and Cell Biology, School of Veterinary Medicine, University of California, Davis, CA 95616, USA

**Keywords:** anaphase B, mitotic motors, spindle elongation, poleward flux

## Abstract

Anaphase B spindle elongation is characterized by the sliding apart of overlapping antiparallel interpolar (ip) microtubules (MTs) as the two opposite spindle poles separate, pulling along disjoined sister chromatids, thereby contributing to chromosome segregation and the propagation of all cellular life. The major biochemical “modules” that cooperate to mediate pole–pole separation include: (i) midzone pushing or (ii) braking by MT crosslinkers, such as kinesin-5 motors, which facilitate or restrict the outward sliding of antiparallel interpolar MTs (ipMTs); (iii) cortical pulling by disassembling astral MTs (aMTs) and/or dynein motors that pull aMTs outwards; (iv) ipMT plus end dynamics, notably net polymerization; and (v) ipMT minus end depolymerization manifest as poleward flux. The differential combination of these modules in different cell types produces diversity in the anaphase B mechanism. Combinations of antagonist modules can create a force balance that maintains the dynamic pre-anaphase B spindle at constant length. Tipping such a force balance at anaphase B onset can initiate and control the rate of spindle elongation. The activities of the basic motor filament components of the anaphase B machinery are controlled by a network of non-motor MT-associated proteins (MAPs), for example the key MT cross-linker, Ase1p/PRC1, and various cell-cycle kinases, phosphatases, and proteases. This review focuses on the molecular mechanisms of anaphase B spindle elongation in eukaryotic cells and briefly mentions bacterial DNA segregation systems that operate by spindle elongation.

## 1. Introduction and Historical Perspective

**Figure 1:**
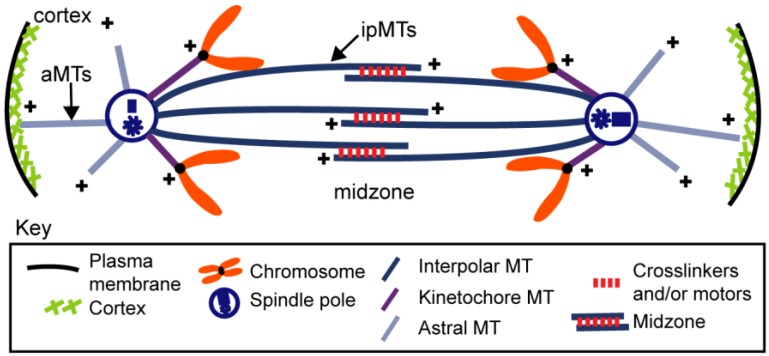
Basic structure of the anaphase B spindle. The major components driving anaphase B spindle elongation are shown, namely ipMTs and the spindle midzone as well as aMTs and the cell cortex, and the structural polarity of spindle MTs is indicated by marking their plus ends. For simplicity, branched augmin-nucleated and chromatin-nucleated MTs that do not reach the poles, as well as pole-nucleated MTs that do not reach kinetochores or the midzone, are not included. Also, anastral spindles lacking centrosomes at the poles are not represented here.

**Figure 2:**
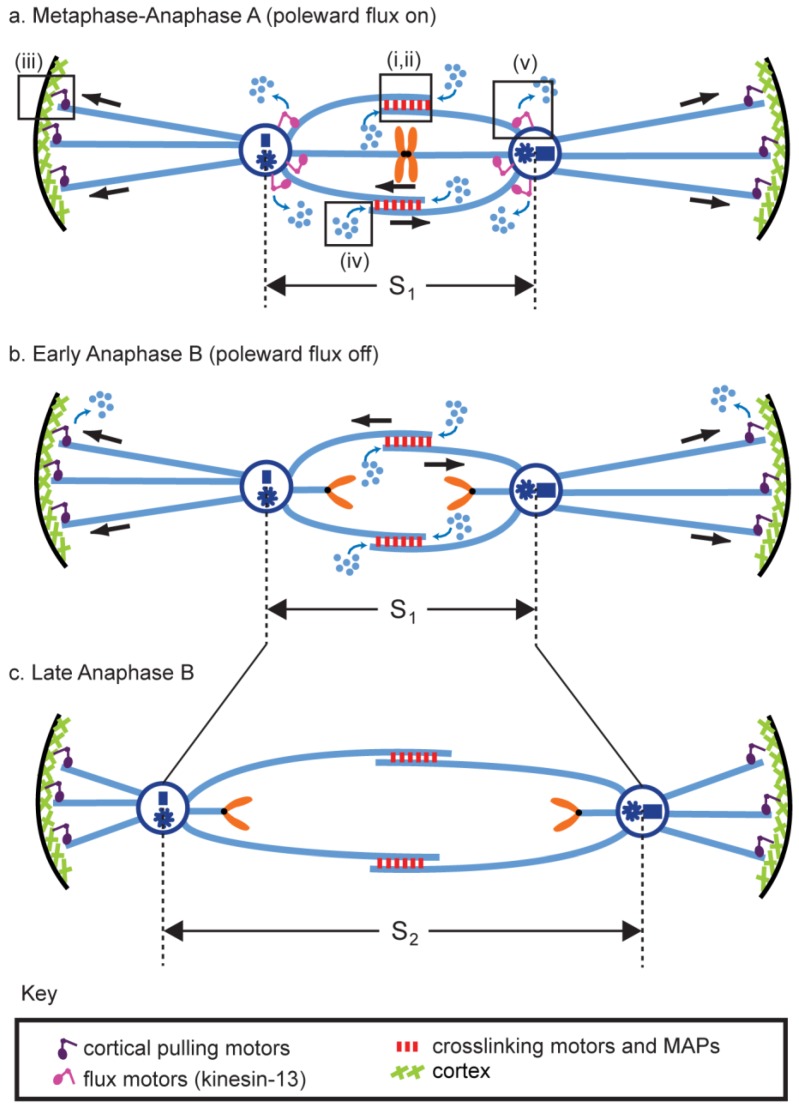
Anaphase B in an idealized and simplified mitotic spindle. The spindle is depicted (**a**) during metaphase-anaphase A (aka pre-anaphase B), when poleward flux is “on” maintaining the spindle at a constant length, S_1_; (**b**) at the start of anaphase B, when flux is turned off so that the spindle can begin to elongate; and (**c**) at late anaphase, when the spindle has completed its elongation to length S_2_. The major biochemical modules are shown, namely midzone (i) pushing or (ii) braking by MT crosslinkers, particularly kinesin-5 motors and Ase1p MAPs; (iii) cortical pulling by depolymerizing proteins and/or dynein motors attached to the cortex that respectively disassemble or translocate along aMTs to pull them and the attached poles outward; (iv) ipMT plus end dynamics, notably net polymerization; and (v) ipMT minus end depolymerization manifest as poleward flux. In most cells anaphase B starts after anaphase A (as depicted here), but there are exceptions, e.g., in mouse eggs anaphase B precedes anaphase A [42]. Unless otherwise indicated, in this and all other figures, arrows depict direction of movement of ipMTs and aMTs.

During anaphase, chromosomes are physically separated on the pre-assembled mitotic spindle machinery by a cell type-specific combination of (i) chromosome-to-pole motility (anaphase A) coupled to the pacman- and/or poleward flux-based depolymerization of kinetochore MTs (kMTs) and (ii) spindle elongation (anaphase B) mediated by cortical force generators and/or midzonal MT-MT sliding motors that respectively pull or push apart the spindle poles (Figure 1 and Figure 2) [1,2,3,4,5,6,7]. Anaphase B spindle elongation appears to be broadly deployed among eukaryotes and in some systems, e.g., *S. cerevisiae* cells and early *C. elegans* embryos, it is the major mechanism of chromosome segregation [8,9]. Moreover, in some bacterial cells, mechanisms strikingly similar to eukaryotic anaphase B spindle elongation segregate DNA [10]. Underscoring the significance of the process, anaphase B spindle elongation contributes to the correction of mitotic chromosome attachment errors [11,12,13] and defects in the anaphase B component of chromosome segregation may contribute to human disease—for example a prolonged anaphase B in lymphocytes appears to correlate with an increased risk of cancer [14]. The focus of the current review is on understanding the basic molecular mechanisms of anaphase B spindle elongation. Reviews of aspects of this topic have been published previously e.g., [15,16,17].

Anaphase B was clearly distinguished from anaphase A in the 1940s by Ris, who showed that spindle elongation in insect cells was more sensitive to inhibition by chloral hydrate than was chromosome-to-pole motion, providing evidence that the two components of chromosome segregation are driven by distinct molecular mechanisms [18,19]. However, anaphase B spindle elongation had apparently been described much earlier, for example by Druner, who proposed a midzonal pushing mechanism in 1894 (see [20] p. 22), and Boveri, who proposed a cortical pulling mechanism in 1888 (see [21] p. 41). Subsequent light microscopy studies have documented the kinetics of anaphase B spindle elongation in a variety of eukaryotic cell types (e.g., see Figure 2 in [3]).

**Figure 3:**
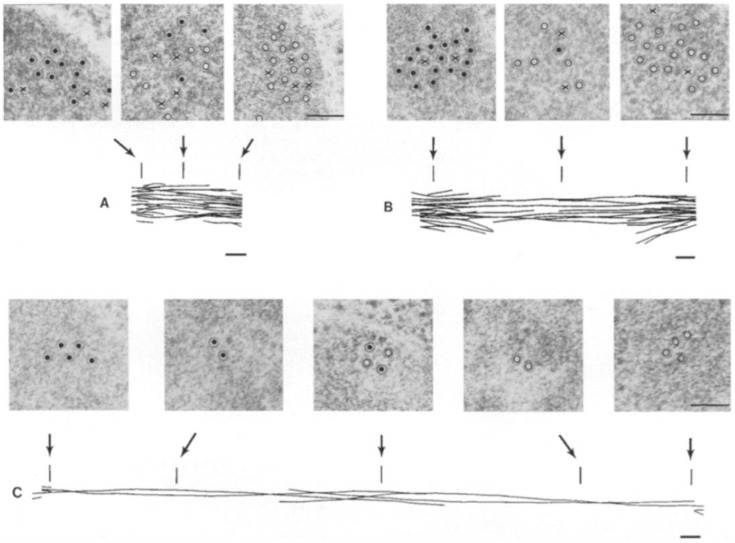
Electron microscopic analysis of anaphase B spindle elongation in budding yeast mitotic spindles showing the structural reorganization of ipMT bundles. 3D reconstructions of (**A**) short spindle; (**B**) early elongating; and (**C**) late elongating spindle. Sample cross sections taken at points indicated by arrows are shown for each reconstruction. In (**B**) kMTs have mostly depolymerized; in (**C**) they have completely depolymerized. Scale bar: 0.1 μm (Originally published as Figure 4 in reference [32], used with permission).

An important advance was the proposal and subsequent testing of a “sliding filament” hypothesis for mitosis [22], in which it was postulated that mitotic motors slide apart adjacent MTs to drive many of the movements of the mitotic spindle that contribute to chromosome movements, in a manner analogous to class-II myosin filaments, which drive the sliding filament mechanism of muscle contraction [23]. Testing the sliding filament model promoted detailed electron microscopy of the organization of mitotic spindle MTs [24,25,26,27] (Figure 3) and a biochemical search for the motors that mediate MT-MT sliding [28,29,30,31].

Electron microscopic analysis of the three-dimensional ultrastructure of the mitotic spindle by McIntosh and colleagues showed that the sliding filament model could not explain all aspects of mitosis e.g., chromosome-to-pole movement during anaphase A, but such a mechanism could drive pole–pole separation during anaphase B spindle elongation [26,32]. This hypothesis was further supported by light microscopic observations of elongating spindles marked by photo-bleaching in living cells [33] and the reactivation of anaphase B spindle elongation in isolated diatom mitotic spindles [34,35]. The inhibition of isolated diatom spindle elongation by a pan-kinesin peptide antibody suggested that a kinesin motor drives anaphase B [36], a hypothesis supported by the characterization of purified kinesin-5 motors with the potential to act like miniature myosin filaments that could cross-link and slide apart antiparallel ipMTs in the spindle midzone [28,37]. While a significant body of evidence supports such a midzone pushing model, other work has shown that the pulling apart of the spindle poles by motors located on the cell cortex can provide an alternative or complementary mechanism to accomplish midzonal MT-MT sliding and anaphase B spindle elongation [38,39,40,41].

Here we review the contribution of these complementary midzone pushing and cortical pulling mechanisms to the sliding filament mechanism of anaphase B spindle elongation in eukaryotic cells (Figure 2). We also briefly mention the bipolar spindle comprising antiparallel bundles of actin-like filaments that elongates by filament polymerization to push apart clusters of R1 plasmids in *E. coli* bacteria, using a mechanism analogous to eukaryotic anaphase B [10].

## 2. Dynamics of Anaphase B in Living Cells

Aspects of mitosis including anaphase B spindle dynamics have been studied using light microscopy for over a century in several systems [43], revealing that, during anaphase B, spindles typically elongate over distances of 1–10 μm at rates of 0.01–0.1 μm/s (Table 1). In favorable cases, anaphase B can be visualized without staining, revealing, for example, that isolated diatom spindles elongate at a rate (0.015 μm/s) [44] approaching that observed in vivo (0.04 μm/s) [45]. Nowadays, however, it is more common to use fluorescence microscopy, e.g., time lapse confocal microscopy of cells containing fluorescent proteins to probe spindle dynamics, e.g., spindle length as a function of time. In *Drosophila* syncytial embryos, for example, about 1000 spindles proceed through mitosis simultaneously in a very well-defined pattern, with anaphase B spindles elongating at a highly reproducible linear rate of ≈0.1 μm/s [46]. Anaphase B spindles in most organisms studied so far elongate at a single linear rate, although some spindles elongate in a biphasic manner at two distinct rates (Table 1). For example, in *Ustilago Maydis*, an initial slow elongation rate is followed by a second faster phase [39], while in *S. cerevisiae* an initial fast phase of spindle elongation is followed by a second slower rate [47].

Interestingly, changes in MT dynamics have been observed at the onset of anaphase B in several systems using FRAP (Fluorescence Recovery after Photobleaching), photoactivation, and FSM (fluorescence speckle microscopy) experiments that monitor fluorescent tubulin behavior. These techniques reveal that spindle MTs display rapid turnover reflecting two types of polymer dynamics coupled to GTP hydrolysis, namely; (i) dynamic instability, characterized by four parameters, the growth rate, shrinkage rate, catastrophe frequency, and rescue frequency; and (ii) poleward flux in which tubulin subunits polymerize at the MT plus ends facing the spindle equator and depolymerize at the minus ends around the poles as the MT polymer lattice slides polewards [48,49,50,51,52,53,54,55]. However, in many systems at the onset of anaphase, spindle MTs display changes in the kinetics of fluorescence recovery in FRAP experiments, reflecting changes in MT dynamics [33,56,57,58,59]. For example, in the fission yeast, *S. pombe*, there is no detectable recovery of fluorescence after photobleaching during anaphase B spindle elongation suggesting a dramatic decrease in MT dynamics [58]. In *Drosophila* embryos, poleward flux within ipMTs stops at the onset of anaphase B [60,61] and ipMT plus ends redistribute to the equator, making the ipMTs more stable [62].

Another method for studying spindle MT behavior utilizes laser ablation experiments to sever one or more MT bundles within the spindle, monitor how the spindle responds, and infer where forces are generated [40,66,67,68]. For example, in *C. elegans* embryos, laser ablation of the spindle midzone causes the poles to move rapidly toward the cell cortex, revealing that the midzone is dispensable for anaphase B spindle elongation and instead acts as a brake [40] due to the braking action of bipolar kinesin-5 motors [64], or the combined action of the MT bundling protein Ase1p/PRC1 and kinesin-6 [69,70]. The role of this braking action is unclear but it may somehow contribute to the fidelity of spindle elongation. In contrast, in the fission yeast spindle, laser dissection showed that midzone pushing is necessary and sufficient for anaphase B spindle elongation [68,71].

## 3. Energetics of Anaphase B

How much force and energy are needed to drive anaphase B spindle elongation at the rates typically observed? It is estimated that very little force, much less than a piconewton (pN), is required to move spindle poles and chromosomes at the speeds observed against cytoplasmic viscous drag [72]. However, it is hard to make precise estimates because the viscosity of cytoplasm is difficult to measure due to its anisotropy and heterogeneity, e.g., [73]. Such a low force value suggests that the free energy released by the hydrolysis of far fewer than 100 ATP fuel molecules could support spindle pole movement over a distance of 10 μm (ignoring imperfections in mechanochemical coupling efficiency and ATP hydrolysis by coupled cell cycle regulatory kinase–phosphatase reactions), which is very small compared to the 10^7^ ATP s^−1^ expended by a “typical” cell (see [74] for rudimentary calculations). On the other hand, famous experiments using calibrated microneedles revealed that insect spindles are capable of exerting far greater forces than this on anaphase chromosomes, approaching a nanonewton (0.7 nN) stall force [75]. The relative contribution of anaphase A and B to this force is unclear, although similar experiments done in echinoderm eggs suggest that the stall force required to specifically inhibit anaphase B spindle elongation is of a comparable magnitude [76]. Indeed, some researchers favor the idea that the force for anaphase A chromosome-to-pole motility could be generated by the same ipMT sliding filament mechanism that elongates the anaphase B spindle via passive crosslinks between the moving ipMTs and adjacent kMTs as discussed by [1]. Furthermore, the inhibition of bipolar kinesin-5 motors sometimes leads to defects in anaphase A as well as anaphase B, suggesting that they could participate in such a crosslinking mechanism [77]. Despite the anaphase spindle’s capability for generating such a high stall force, it has a much lower specific power output than e.g., muscle or motile cilia, plausibly reflecting its adaptation to precision rather than power [78].

Some of the most direct experiments on ATP expenditure by the spindle during anaphase B have utilized in vitro cell models. For example, in permeabilized vertebrate cultured cells where midzone pushing and cortical pulling may cooperate, anaphase B spindle elongation, unlike anaphase A, requires ATP hydrolysis (half-maximal rate at ≈100–200 μM MgATP), whereas other nucleotides such as GTP cannot substitute for ATP [79]. Isolated diatom central spindles supplied with ATP fuel will elongate at a constant linear rate that is independent of tubulin polymerization which influences the extent but not the speed of elongation [44]. At least a thousand-fold less force (≈1 fN) is needed to elongate these spindles against viscous drag at the rates observed in vitro compared to in vivo because pole motility is opposed only by water (an isotropic liquid), whose viscosity is much lower than that of cytoplasm [44]. Consequently, the free energy of hydrolysis of a single ATP molecule can provide more than enough energy, with the fuel very likely being used by some type of kinesin motor [36]. Given the low ATP turnover involved, the suggestion that a striated muscle-type ATP-regenerating creatine kinase/phosphocreatine/ADP system plays a significant direct role in anaphase B spindle elongation may merit re-evaluation [80,81]. The reactivation of anaphase B in echinoderm eggs is different in that it requires GTP but not ATP, and the presence of assembly-competent tubulin affects both the rate and extent of elongation, suggesting a dominant role for MT polymerization [82]. Therefore potential force generators for anaphase B in different systems include not only MT-based motors like kinesins and dyneins, but also dynamic cytoskeletal filaments that can polymerize or depolymerize to exert pushing and pulling forces, respectively, and midzonal MT-crosslinking MAPs, which have recently been proposed to exert entropic expansion forces [83,84,85].

## 4. Structural Studies of the Anaphase B Spindle

Classic work using painstaking electron microscopy (EM) has elucidated the three-dimensional organization and polarity patterns of MTs within the mitotic spindles of several cell types, providing an important foundation for understanding the mechanism of anaphase B spindle elongation, as well as other aspects of mitosis (Figure 3). For example, early serial section electron microscopic analysis of human cultured cells during anaphase and telophase was consistent with the hypothesis that ipMT bundles consist of two sets of MTs that emanate from opposite poleward regions and overlap at the spindle midzone [27]. In some cases the polarity patterns of MTs within such ipMT bundles have been directly determined using the method of hook decoration [86]; for example, in endosperm cells of the plant *Haemanthus* that were fixed during anaphase, the two sets of opposite polarity ipMTs were shown to be oriented with their minus and plus ends facing the spindle poles and the midzone, respectively [24]. Although the number of ipMTs varies from less than 10 in budding yeast to hundreds in diatoms and cultured cells, the same overall structural organization is thought to apply to ipMT bundles within most spindles (Figure 1). This has been confirmed for budding yeast [32], fission yeast [87,88], and diatom [26], for example, where the minus ends of the overlapping ipMTs appear to physically interact with the spindle poles. The outward sliding of these ipMTs could therefore directly exert compressive forces on the poles to push them apart, leading to anaphase B spindle elongation. This structural organization further suggests that a plus end-directed antiparallel ipMT-crosslinking motor located in the midzone, with a functional organization equivalent to a bipolar myosin-II filament [89], could perform such a function e.g., see Figure 4b in [28]. The hypothesis that motors associated with interdigitating antiparallel ipMTs at the anaphase B spindle midzone could push apart the spindle poles in diatoms is supported by studies of the ATP-dependent reactivation of the elongation of isolated central spindle preparations [35]. Like the anaphase spindles seen in intact diatom cells, by EM these isolates display a robust midzone of ≈600 overlapping ipMTs that slide apart following ATP addition to drive pole–pole separation [34].

A somewhat different picture emerged from the detailed EM analysis of ipMT bundles during anaphase in PtK1 cells [90]. These bundles are also organized into two sets of opposite polarity with their plus ends overlapping at the midzone, but the minus ends of most of these ipMTs do not actually reach the poles, suggesting that they cannot directly push on the poles to drive spindle elongation. While it is possible that these ipMTs act indirectly, e.g., via an interaction between their minus ends and kinetochore MTs whose minus ends do contact the poles, it is perhaps more plausible to think that, in this system, the poles are pulled apart by a cortical pulling mechanism [90]. In this scenario it is possible that bipolar MT crosslinking motors within the spindle midzone could serve as brakes that restrict the rate of ipMT-MT sliding [64,65], thereby enhancing the fidelity and directionality of pole–pole separation [1].

**Figure 4:**
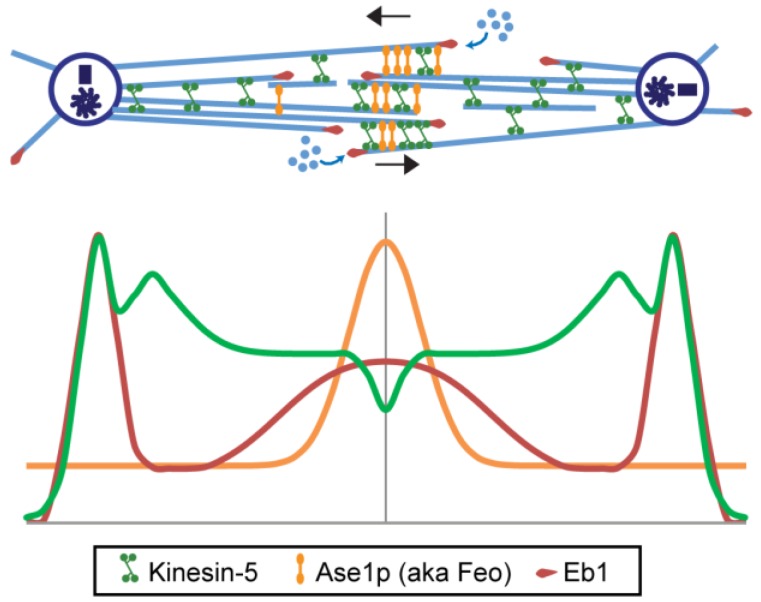
Anaphase B spindle in a *Drosophila* embryo. The upper drawing of the anaphase B spindle has a pole–pole axis corresponding with that of the drawing below showing the relative distribution of kinesin-5 motors, Ase1p crosslinkers, and the plus end binding protein Eb1 along the spindle (adapted from [93]).

The use of EM to directly visualize the mitotic motors that are predicted to crosslink and slide apart, or constrain the sliding apart, of antiparallel ipMTs in the spindle midzone has proven difficult and has yielded less definitive information. MT-MT cross-bridges have been seen in EM images of sectioned mitotic spindles but they are sometimes rather ill-defined, vary in average length from about 20 nm to about 60–65 nm, usually do not display an obvious regular axial spacing, and sometimes appear to form a “matrix” [25,26,32,37,87,91]. It is plausible to think that these cross-bridges could comprise non-motor MAPs such as Ase1p (aka PRC1), or mitotic motors such as the bipolar kinesin-5 (discussed in the next section). Consistent with the latter idea, for example, light microscopy of both living and fixed *Drosophila* embryo anaphase spindles suggests that kinesin-5 localizes along the entire length of ipMT bundles where it co-localizes via competitive binding with Ase1p at the spindle midzone (Figure 4) [37,92,93]. In this system, serial section EM is consistent with the basic conclusion of the earlier pioneer work [27] in suggesting that each of the anaphase B spindle’s nine ipMT bundles contains about 30–40 MTs per half-spindle that are parallel near the poles and overlap in an antiparallel orientation for ≈2–3 μm at the midzone [37]. By immuno-EM, Au-coupled anti-kinesin-5 clearly decorated MTs all along these bundles and was sometimes seen to be associated with 60–65 nm long cross-bridges between adjacent MTs [37], a length similar to the 57–61 nm length of the purified *Drosophila* kinesin-5 rod [28,94]. While these results are consistent with the idea that kinesin-5 could form at least some of the cross-bridges seen in EM sections of ipMT bundles, they do not prove it and more definitive and comprehensive information concerning the identity and architecture of these structures would be useful, especially given the complex molecular composition of the spindle midzone [95,96]. This important, challenging problem merits further work.

## 5. Conserved Biochemical Modules Involved in Anaphase B

**Figure 5:**
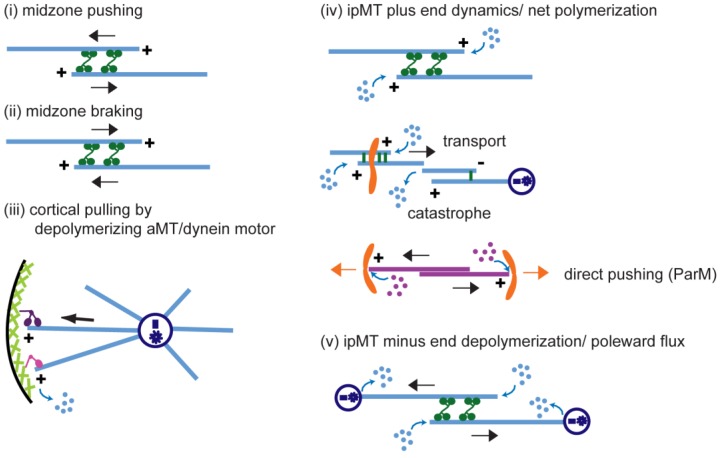
Anaphase B modules. The five biochemical modules depicted in Figure 2 (modules **i–v**) that are deployed to various extents in different systems are shown in more detail.

Current evidence suggests that a handful of conserved biochemical “modules” are deployed to different extents in a combinatorial fashion in distinct cell types to accomplish the elongation of the anaphase B spindle, including outward sliding of antiparallel ipMTs to push apart spindle poles, restriction of ipMT sliding by midzonal crosslinkers, growth of ipMT plus ends by MT polymerization, cortical forces that pull the spindle poles outwards and the use of poleward MT flux as a regulatory switch (Figure 5).

### 5.1. (Module i) Midzone Pushing: Pole–Pole Separation by Outward Sliding of Antiparallel ipMTs

Much of the work described in section 10.4 supports the idea that plus-end-directed bipolar mitotic motors could act at the spindle midzone to slide apart overlapping ipMTs and generate pushing forces to separate the anaphase B spindle poles. This model is especially appealing in the case of the diatom central spindle [26,35], where it was further supported by observations that the laser microbeam-induced destruction of ipMTs at the presumptive site of force generation in the spindle midzone, but not around the poles, inhibited spindle elongation [67]. Similar results supporting an ipMT pushing mechanism have been obtained using laser microsurgery of elongating fission yeast spindles [68,71]. In living, cultured PtK1 cells, the dynamics of anaphase B spindles containing fluorescent tubulin and marked by photobleaching was studied using light microscopy, leading to proposals that the sliding apart of ipMTs by a force generated at the zone of interdigitation at the midzone could contribute to spindle elongation [33] (although subsequent EM of these spindles yielded the caveats noted above; [90]). Circumstantial evidence in support of a bipolar kinesin-5-mediated midzonal ipMT-MT sliding model was obtained in *Drosophila* embryos where the spindle poles separate during anaphase B at a linear rate of ~0.1 μm/s, which, as expected, is almost exactly twice the rate at which tubulin speckles flux towards the opposite poles along ipMTs (0.05 μm/s) during pre-anaphase B and twice the speed at which purified fly embryo kinesin-5 moves MTs in motility assays (0.05 μm/s) [31,77]. We discuss the properties of the presumptive motors in more detail below.

### 5.2. (Module ii) Midzone Braking

MT-MT crosslinking MAPS and motors on the spindle midzone can also serve as brakes that restrict the rate and extent of pole–pole separation driven by antagonistic force generators e.g., cortical pulling motors [64,65,96,97,98,99].

### 5.3. (Module iii) Cortical Pulling Apart of the Anaphase B Spindle Poles

One interpretation of the EM work cited above, showing that the minus ends of ipMT bundles in PtK1 cells do not appear to reach the spindle poles [90], is that a mechanism other than ipMT mediated pushing forces could operate to separate the spindle poles in some cells, for example a force generator that acts at the cell cortex to exert pulling forces on the asters to pull apart the associated spindle poles [38]. Indeed, a significant body of evidence supports the existence of such external pulling forces that pull on the spindle poles to control pole–pole spacing and even to position the entire spindle [40,100,101]. Candidates for such force generators include cortically-anchored dynein or astral MT depolymerization, with kinesin-5 acting either as a supplementary pole-separating force generator or as a counteracting brake [39,64,102]. We assume here that direct contacts between aMTs and the cell cortex are required to exert pulling forces on the spindle poles [103] but it should be noted that cytoplasmic force generators may also somehow be able to pull asters outward in the absence of cortices e.g., [104].

### 5.4. (Module iv) ipMT Plus End Dynamics and Net Polymerization

MT polymer dynamics, characterized by dynamic instability and poleward MT flux, obviously play critical roles throughout mitosis [48,49]. For example, during anaphase B in some systems, overlapping ipMTs at the spindle midzone grow by polymerization of their plus ends as they slide apart. This was a conclusion of the clever photo-bleaching experiments done on elongating anaphase B spindles in PtK1 cells by Saxton and McIntosh [33], in which tubulin subunits were observed to add on to the plus ends of the outwardly sliding ipMTs. Further support for this idea was obtained using isolated diatom spindles that, when supplied with ATP fuel in the absence of free tubulin subunits, will elongate to an extent that is limited by the size of the original overlap zone [34,35]. However, when tubulin subunits are added to these preparations, the subunits polymerize onto the plus ends of pre-existing ipMTs causing the length of the initial overlap zone and the extent of subsequent ATP-induced spindle elongation to increase by a corresponding amount [105,106]. Thus the polymerization–depolymerization of ipMT plus end at the spindle midzone appears to play an important role in determining the extent of sliding apart of antiparallel ipMTs at the midzone and, in turn, the extent of anaphase B spindle elongation. In *Drosophila* embryo spindles, a spatial gradient of MT catastrophe frequencies (decreasing towards the equator) is established at the onset of anaphase B, causing ipMTs to polymerize at their plus ends and grow at the equator to invade the midzone [62]. A complementary mechanism occurs during early anaphase in cultured human cells, where augmin/γ-TuRC nucleates the branching polymerization of ipMT plus ends on pre-existing spindle MTs [107]. In both cases the growing ipMTs can then be crosslinked by MAPs and motors around the equator to produce a more robust anaphase spindle midzone, but the significance of this for the dynamics of spindle elongation remains to be determined.

A variant of the coupling of ipMT plus end polymerization to ipMT sliding occurs in some prokaryotes where polymerization generates compressive forces that directly push apart the poles. Bacterial cells are generally thought to lack filament-sliding motors analogous to kinesins, dyneins, and myosins, and in the R1 plasmid segregation system discussed in Section 6.3, for example, the bidirectional polymerization of antiparallel cytoskeletal filament bundles can directly generate forces that drive spindle elongation [10]. Interestingly, recent work suggests that an analogous pushing mechanism based on midzonal ipMT plus end polymerization may contribute, in a redundant fashion, to spindle elongation and chromosome segregation in *C. elegans* embryos [108,109].

Another biochemical mechanism based on ipMT plus ends dynamics, named “slide-and-cluster” has been proposed to control spindle length in *Xenopus* extract spindles [110]. Here, antiparallel MTs nucleated on chromatin that grow by plus-end polymerization, are slid apart, minus-end leading, by kinesin-5 motors, with further poleward transport of the MTs being facilitated by minus-end-directed motors that move them along pre-existing MT tracks. The dynamics of the sliding MT plus ends determines their lifetime since they can disappear via catastrophic depolymerization so that spindle length depends on both the lifetime and the rate of poleward MT transport of these spindle MTs. Whether this module contributes to anaphase B, something that is difficult to test in extracts, is discussed later (see theoretical models).

### 5.5. (Module v) ipMT Minus End Depolymerization: Poleward Flux as a Regulatory Switch for Anaphase B

It has long been recognized that the flux of tubulin towards the spindle poles is a striking feature of many mitotic spindles [50,53]. While most attention has focused on its role in anaphase A chromosome-to-pole motility, it may also play a critical regulatory role in chromosome segregation by turning on and off anaphase B spindle elongation, at least in some systems. In *Drosophila* embryo mitotic spindles, for example, there is evidence that the suppression of poleward tubulin flux within ipMT bundles due to the inhibition of ipMT minus end depolymerization can initiate and control the rate of anaphase B spindle elongation [60,77], but whether this is a system-specific or broadly utilized mechanism remains to be established.

### 5.6. Combination of Modules and the Force Balance Concept

Spindles in different cell-types utilize different combinations of antagonist or complementary modules to produce or modulate the force that drives anaphase B spindle elongation. For example, the net polymerization of ipMT plus end polymerization (module iv) can complement midzone pushing (module i) to enhance the extent of spindle elongation [105] whereas midzone braking (module ii) can antagonize cortical pulling (module iii) to slow down the rate of spindle elongation [64]. Importantly, anaphase B can be controlled by antagonistic modules that create a force balance of the type initially proposed by Ostergren to control metaphase chromosome position [111,112], as reviewed in Chapter 4. A good example of this was mentioned in the previous paragraph. The combination of midzone pushing (module i) and ipMT depolymerization (module v) during pre-anaphase B produces a force balance in which ipMTs undergo poleward flux and the spindle is maintained at a constant steady state length. When this force balance is tipped at anaphase B onset by the inhibition of ipMT minus end depolymerization at spindle poles, the opposing forces become unbalanced, allowing midzone pushing to exert net outward force on the spindle poles to elongate the anaphase B spindle [60,61,113].

## 6. Properties and Functions of the Molecular Nuts and Bolts of the Anaphase B Machinery

The anaphase B spindle is thought to comprise fairly “typical”, structurally polar MTs that are assembled from αβ-tubulin dimers in a head-to-tail fashion with the β subunits facing the MT plus ends and that display both dynamic instability and poleward flux [48,49,50,114]. A full understanding of the mechanism of anaphase B requires an elucidation of the functions and mechanism of action of all the molecules that interact with these MTs, but in many cases this is difficult because the functional perturbation of key molecules can also interfere with earlier phases of mitosis, thereby obscuring later roles in anaphase B. This is compounded by the well-known existence of functional redundancy between different mitotic mechanisms [115], combined with the fact that many key molecules localize to multiple sites in the spindle e.g., to the kinetochore, midzone, and cortex, making their site of action in anaphase B difficult to discern.

### 6.1. Molecules of the Central Spindle

#### 6.1.1. Antiparallel ipMT-Crosslinking MAPs of the Ase1p Family

The anaphase B-specific functions of homodimeric MT-MT crosslinking MAPs of the Ase1p family (i.e., anaphase spindle elongating protein 1; aka PRC1, MAP65, Feo) are unusually obvious because these MAPs only localize to the spindle midzone following metaphase and their function is required for anaphase B spindle elongation [116,117]. Three properties of Ase1p MAPs underlie their critical role in organizing the midzone and facilitating anaphase B spindle elongation. First, Ase1p dimers preferentially crosslink ipMTs into antiparallel orientations where they oligomerize to form a “matrix” between pairs of bundled MTs [84,117,118]. This matrix may correspond to the “osmiophilic matrix” seen in EMs of stained diatom spindles [26] (reviewed in [96]). This bundling of MTs by Ase1p may be facilitated by its structure—it has been proposed that Ase1p is a flexible molecule in solution, which adopts a more rigid conformation only when bundling antiparallel MTs [119]. Second, the resulting Ase1p complex can serve as a key “regulatory hub” for controlling the cell-cycle-dependent localization of various motors and other proteins to the midzone in a system-specific fashion (reviewed in [17]). For example, at anaphase onset in budding yeast, the cell-cycle-regulated de-phosphorylation of Ase1p allows it to recruit kinesin-5 sliding motors to the midzone to drive spindle elongation [120] whereas in *Drosophila* embryos the Ase1p family member, Feo, partially restricts the association of kinesin-5 sliding motors to the anaphase B spindle midzone (Figure 4) [93]. In the latter system, the dissociation of Ase1p from the midzone permits more kinesin-5 to bind in its place, but anaphase B spindle elongation is then impaired, suggesting that the Ase1p-mediated spindle midzone organization is required to facilitate the kinesin-5-mediated ipMT sliding filament mechanism that underlies anaphase B [93]. In light of these results, it is tempting to speculate that the kinesin-anchoring midzone matrix associated with anaphase B in Diatoms may comprise Ase1p [121]. Finally, striking new work suggests that diffusible Ase1p crosslinkers can also directly generate forces for ipMT-MT sliding via an ATP hydrolysis-independent entropic expansion mechanism that could, for example, control the length of the antiparallel overlaps at the midzone during anaphase B spindle elongation [85,122].

In the context of midzone organization by an Ase1p “matrix,” it is worth noting that the role of a spindle matrix distinct from MT, MAPs and MT-based motors but capable of augmenting the activities of these well-characterized spindle components during mitosis continues to draw attention e.g., [123]. However, apart from the aforementioned work on Ase1p, we are not aware of any evidence that such an entity operates during anaphase B spindle elongation. For example, in *Drosophila* embryos, a lamin B spindle envelope that has been proposed to form a matrix that augments the activities of mitotic motors during earlier phases of mitosis is disassembled prior to anaphase B onset [124].

#### 6.1.2. MT Crosslinking and Sliding Motors; Kinesin-5 Plus Kinesins-4, -6, -8, and -12

Once ipMTs have been organized into an ordered array of antiparallel bundles at the midzone by Ase1p crosslinkers, they can be slid apart by various combinations of ipMT-MT crosslinking and sliding motors, most notably kinesin-5. Purified kinesin-5 motors display a bipolar, homotetrameric ultrastructure consisting of pairs of motor domains at opposite ends of a central 60 nm long rod [28,31,94]. A novel four-helix bundle called the “BASS” (or Bipolar ASSembly) domain, comprising a pair of intertwined antiparallel coiled-coil dimers stabilized by patches of hydrophobic and charged residues, directs the assembly of four motor subunits into these bipolar tetrameric minifilaments [94,125], whose four motor domains can move slowly and moderately processively towards the plus ends of MTs against substantial opposing forces [126,127,128]. This unique homotetrameric architecture is essential for kinesin-5 function during mitosis [129] plausibly because it allows kinesin-5 motors to dynamically interact with spindle MTs via a reaction-diffusion mechanism [92], preferentially binding MTs in the antiparallel orientation [130], and driving or constraining their sliding apart throughout mitosis by a sliding filament mechanism [30,98]. Kinesin-5 was discovered based on its essential role in early bipolar mitotic spindle assembly or maintenance [126,131] but subsequent work in several systems also supports a role in driving outward ipMT sliding and spindle elongation during anaphase B [77,132,133,134,135]. In some cases, however, kinesin-5 appears to serve as a brake that restricts spindle elongation [64,65,97,99]. Cutting-edge optical trap motility assays provide mechanistic insights into how bipolar kinesin-5 motors can switch between the generation of both outward sliding and inward braking forces on crosslinked MTs [98]. It should also be noted that budding and fission yeast kinesin-5 motors are capable of reversing their polarity of MT-based motility [136,137,138], but whether this is significant for the mechanism of anaphase B is, to our knowledge, unknown.

Members of the kinesin-4, -6, -8, and -12 families of plus-end-directed motors could also contribute to ipMT-MT crosslinking and sliding during anaphase B. For example, the kinesin-4 KLP3A organizes midzonal ipMT bundles in fly spindles and somehow couples the downregulation of poleward flux to the onset of spindle elongation at anaphase [60,139]. Kinesins-6 and -8 have been reported to mediate antiparallel MT-MT sliding based on in vitro assays [140,141] and they could therefore augment or replace the function of kinesin-5 in driving anaphase B spindle elongation. Kinesin-8 is best known as a MT-translocating, length-dependent MT depolymerase [142], which influences spindle assembly and length control throughout mitosis. In budding yeast, for example, a complex interplay between its MT-depolymerizing and MT-MT sliding activity appears to contribute to both spindle elongation and disassembly during anaphase B [141,143]. Kinesin-6 dimers, on the other hand, often co-assemble with two subunits of a G-protein cofactor, containing a GTPase-activating domain for Rho-family GTPases (RhoGAP domain), which, in contrast to the prevailing view [144] was recently reported to be dispensable for MT-MT bundling but is required for MT motor activity [145]. The resulting heterotetrameric complex is usually thought to organize the anaphase spindle midzone to control normal cleavage furrow assembly and cytokinesis [95,146]. In fission yeast, however, kinesin-6 is proposed to form homotetramers, based on chemical crosslinking, that bind Ase1p in a phosphorylation-dependent manner to form a complex that interacts with and slides apart antiparallel ipMTs at the midzone to drive anaphase B spindle elongation [147]. Finally vertebrate kinesin-12 can crosslink and slide adjacent MTs in vitro [148] and is a candidate motor driving spindle elongation during *C. elegans* meiosis [149] but it preferentially crosslinks MTs into the parallel rather than antiparallel orientation and, although it substitutes for kinesin-5 function during spindle assembly, to our knowledge, a role in anaphase B has not been reported.

It is perhaps worth emphasizing that many MT crosslinking kinesin motors that organize and control the length of the spindle midzone also play key roles in organizing the cleavage furrow for cytokinesis (although the specific roles of some of them in anaphase B spindle elongation e.g., kinesin-4 recruited to the midzone by Ase1p/PRC1 [150,151,152,153] requires clarification). The role of midzonal kinesins in cytokinesis, which is not a focus of the current manuscript, has been well covered in a recent review [146].

#### 6.1.3. Molecules Controlling MT Plus end Dynamics

A complex network of MT plus end tracking proteins (+TIPs) probably plays important roles in controlling ipMT plus end dynamics at the spindle midzone during anaphase B, but this is a topic that requires further work. +TIPs that enhance MT plus end polymerization include autonomous MT binding proteins such as the master regulator, EB (end binding) protein, and Tog-domain XMAP215 proteins, along with the “hitchhikers” that they recruit to MT plus ends such as CLASP, another Tog protein, whereas proteins such as kinesin-13 and kinesin-8 antagonize these proteins by depolymerizing MT plus ends [114]. Impressive biochemical reconstitution experiments are being used to study these complexes [154] and evidence supporting their possible role in anaphase B emerges from observations that in *C. elegans* zygotes and mammalian cells, for example, CLASPs localize to the anaphase spindle midzone where they may promote ipMT polymerization during spindle elongation [155,156]. Moreover, based on the effects of CLASPs on kMT dynamics in *Drosophila* cells, it is plausible to think that +TIPs could promote ipMT plus end polymerization to contribute to poleward flux during pre-anaphase B when the spindle maintains a constant steady state length, as well as to anaphase B spindle elongation [157], but this requires testing. Also requiring further functional analysis are the results of in vitro assays which suggest that bipolar kinesin-5 motors not only slide apart ipMTs at the midzone but may also contribute to +TIP activity by stimulating the polymerization of ipMT plus ends at the midzone [158]. In *Drosophila* embryos, the transition from poleward flux to spindle elongation is accompanied by the rapid formation of a spatial gradient of MT plus end catastrophe events, decreasing in the anti-poleward direction, which causes ipMT plus ends to grow towards the equator and augment the midzone where outward ipMT-MT sliding forces are generated [62]. In human cells the augmin/γ-TuRC complex nucleates the branching polymerization of MTs to promote robust central spindle assembly [107].

Another possible mechanism for assembling MT plus ends at the midzone may merit investigation. The anterograde heterotrimeric kinesin-2 motor [159] is understood to deliver tubulin subunits to the MT plus ends of growing ciliary axonemes [160], but it has also been localized to the midzone of anaphase sea urchin embryos, where its role is unknown [161]. It is tempting to speculate that kinesin-2 may also deliver tubulins for assembly at the plus ends of overlapping ipMTs in the anaphase spindle midzone, but this idea has not, to our knowledge, been tested so far.

#### 6.1.4. Molecules Controlling ipMT Minus End Dynamics

A topic that merits further work is how ipMT minus end dynamics contribute to anaphase B, especially at the poles, where compressive forces are exerted [114]. One well-established minus-end regulator, γ-TuRC is understood to nucleate MT polymerization at centrosomes, but it can also bind MT minus ends throughout the spindle whereupon a slide-and-cluster mechanism (which could contribute to anaphase B spindle elongation—see theoretical models) transports the MTs to the poles [162]. There is better evidence that minus-end-targeting −TIPs such as CAMSAPs and Patronin [114] play important roles in anaphase B, at least in some systems. These proteins stabilize MT minus ends against the depolymerizing activity of kinesin-13 [163,164,165] and in *Drosophila* embryos, the Patronin-mediated inhibition of kinesin-13-dependent ipMT minus end depolymerization at the poles occurs in response to cyclin B degradation [113,166]. This induces a switch from poleward flux to anaphase B spindle elongation by allowing outwardly sliding ipMTs to push apart the spindle poles, but whether this switch is used elsewhere is unknown.

#### 6.1.5. Chromosomal Proteins Required for Anaphase B Spindle Elongation

An important and sizeable set of proteins known as chromosomal passenger proteins translocate from the chromosomes to the spindle midzone at anaphase onset and are understood to perform key roles in coordinating progress through anaphase B, for example by stabilizing the spindle midzone and recruiting proteins required for telophase, cytokinesis and mitotic exit [167]. In *C. elegans* embryos, it was recently reported that a subset of kinetochore proteins is required for spindle midzone assembly and normal anaphase B spindle elongation [156]. Sorting out the precise relationships between these chromosomal proteins and anaphase B is a cutting-edge problem in mitosis research.

### 6.2. Molecules of the Cortical Pulling Machinery

#### 6.2.1. Attachment of MT Plus Ends to the Cortex

The cortical pulling mechanism (Figure 2 and Figure 5 (iii)) requires that astral MT plus ends interact with the cell cortex, and this is again mediated by the +TIP network, which includes MT-depolymerases e.g., kinesin-13, MT polymerases, e.g., CLASPs, and motors, most notably dynein-dynactin, some of which interact directly with the lipid bilayer but usually bind via membrane-associated adaptors or via the cortical actin cytoskeleton [114].

#### 6.2.2. Cortical Force Generators

The spindle poles can potentially be pulled apart by; (a) a polymer ratchet mechanism in which the plus ends of astral MTs linked to the cell cortex depolymerize to pull the poles outward [49,102]; and (b) a motor-dependent mechanism in which dynein anchored at the cell cortex walks towards the minus ends of astral MTs to pull the poles outward [168,169]. For example, observations and simulations of astral MT depolymerization following contact of their plus ends with the *C. elegans* embryo cortex suggest that cortical adaptors may couple aMT disassembly to the generation of forces for pulling apart the poles [103]. Moreover, single-molecule TIRF microscopy in fission yeast cells has allowed the direct visualization of dynein dynamics as it diffuses in the cytoplasm, attaches to an aMT, undergoes 1-D diffusion along the aMT to reach the plus end, whereupon it “off-loads” and binds to the cell cortex [170]. Once anchored at the cell cortex, dynein may pull the spindle poles outward in two ways, first by using ATP hydrolysis to walk towards the minus ends of the aMT [168] and second, based on clever in vitro experiments, by inducing the catastrophic depolymerization of captured aMTs to generate pulling forces on the spindle poles [171]. The resulting pulling forces may then participate in many aspects of mitosis and mitotic spindle positioning e.g., [172,173] including anaphase B spindle elongation [38,39].

### 6.3. Molecules Involved in Prokaryotic Anaphase B

Understanding of prokaryotic DNA segregation by the bacterial cytoskeletal machinery has advanced enormously in the past two decades. It is now thought that, in different bacteria, at least three types of dynamic polymer can mediate this process. These polymers are assembled from the actin-like protein ParM, the ATPase ParA, or the tubulin-like protein, TubZ [174]. Of these, ParM filaments appear to segregate R1 plasmids in *E. Coli* by an antiparallel array of filaments that resembles the overlapping MTs of the spindle midzone that participate in eukaryotic anaphase B [10]. Briefly, bundles of ParM filaments use ATP hydrolysis to undergo dynamic instability, allowing them to search and capture the centromeres (*parC*) of “sister” plasmids harboring bound ParR adaptor protein, and to push the captured plasmids to opposite ends of the bacterial cell before depolymerizing again. Although the left-handed parallel two-stranded ParM filament is structurally polar, the two ends elongate at equivalent rates, and adjacent filaments display at least a 5:1 preference for pairing in the antiparallel orientation in vitro [175]. A striking reconstitution of the R1 plasmid segregation system has been accomplished, in which ParM filaments polymerize bidirectionally to push apart ParR/*parC* coated beads [176].

## 7. Cell Cycle Control of Anaphase B

The cell cycle control of anaphase B spindle elongation has been reviewed recently by [17] and subsequent work suggests that this is likely to be complex [177]. Briefly, it is understood that anaphase onset occurs following the dephosphorylation of cyclin dependent kinase (cdk1) substrates leading to inactivation of the spindle assembly checkpoint (the SAC, as reviewed in chapter 5, which ensures proper metaphase chromosome alignment) and the loss of cohesion between sister chromatids that can then separate and move poleward [178,179]. In animal cells, the sequential ubiquitin-dependent proteolytic degradation of distinct mitotic cyclins is required for progression through mitosis, with cyclin A degradation allowing progression through metaphase and cyclin B degradation, which occurs immediately after the SAC is satisfied, being necessary for anaphase B [179]. A third cyclin B3 has been found in *Drosophila* whose destruction follows that of cyclin B to promote anaphase B spindle elongation [180,181].

In many organisms, anaphase A and B start simultaneously after chromatids disjoin, i.e., kMT shortening moves chromosomes poleward and the spindle elongates at the same time. In *Drosophila* embryos, however, there is a distinct transition between anaphase A and anaphase B which is regulated by cyclin B degradation [62]. In this system the patronin-dependent downregulation of poleward flux is proposed to function as a regulator of the onset and the rate of anaphase B [60,61,62,113]. As emphasized by [17], the MT bundler Ase1p serves as a regulatory hub for anaphase B in many model organisms. Ase1p accumulates at the anaphase spindle midzone in a phosphorylation- and motor-dependent manner, where it not only controls midzone organization via MT-MT crosslinking but also recruits other key anaphase B proteins. For example, in yeast and *Drosophila* embryos, Ase1p mediates the recruitment of several ipMT sliding motors and this activity is required for proper spindle midzone organization and elongation during anaphase B [93,120,147].

Thus, the regulation of anaphase B spindle elongation is complex. It occurs at several levels and involves ubiquitin-dependent proteolytic degradation, cdk and phosphatase-dependent phosphorylation-dephosphorylation cycles, the key midzone regulatory MAP, Ase1p, as well as the midzonal motors which localize key molecules to the midzone, and whose localization is, in turn, dependent on the regulatory molecules that they localize. In addition, at least in some systems, the up- and downregulation of poleward flux plays important regulatory roles associated with anaphase B. Moreover, exciting recent work indicates that the control of anaphase B involves the dynamic cooperation and antagonism between functionally interdependent kinases, phosphatases, MAPs and motors capable of producing spatially-controlled feedback loops that coordinate the dynamic turnover of phosphorylation sites to orchestrate spindle midzone assembly and elongation [177,182]. A similar dynamic complexity is likely to control the operation of the cortical pulling machinery involved in anaphase B as well.

## 8. Anaphase B in Model Systems

**Figure 6:**
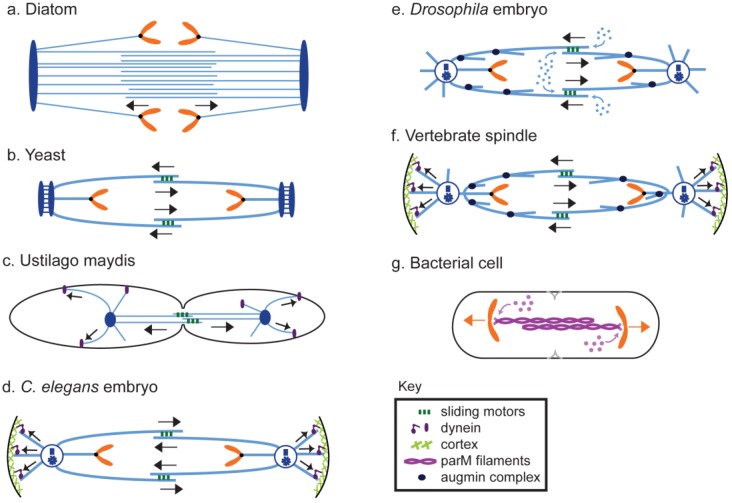
Anaphase B spindle design in different systems. The simplified drawings depict spindles from (**a**) diatoms; (**b**) budding yeast; (**c**) the rust fungus *Ustilago*; (**d**) early *C. elegans* embryos; (**e**) *Drosophila* syncytial embryos; (**f**) vertebrate cultured cells; and (**g**) bacterial cells. Not drawn to scale.

Here we briefly survey how the aforementioned modules and molecules are deployed and combined in a few model organisms to mediate anaphase B (Figure 6 and Table 1). It is likely that the study of “non-mainstream” organisms could further uncover different, more exotic mechanisms of anaphase B spindle elongation [183].

### 8.1. Diatoms

The diatom mitotic spindle (Figure 6a) has been a key model system for understanding the mechanism of anaphase B spindle elongation. It is unusual in having an almost paracrystalline array of hundreds of ipMTs that overlap to form the central spindle which links the spindle poles, and is spatially separated from the kMTs. The hypothesis that the central spindle uses an antiparallel ipMT sliding filament mechanism, coupled to ipMT plus end polymerization, to push apart the spindle poles has gained very strong support from seminal work involving detailed electron microscopy [26], live cell functional perturbation [67] combined with the in vitro reactivation of spindle elongation in isolated central spindles [35]; reviewed by [16]. The observation that pan-kinesin peptide antibodies inhibit spindle elongation in vitro suggests that one of the aforementioned MT-MT sliding kinesins uses ATP hydrolysis to drive ipMT-MT outward sliding in this system [36].

### 8.2. Fungi

#### 8.2.1. Yeast

Anaphase B spindle elongation represents the major mechanism underlying chromosome segregation in *S. cerevisiae* and *S. pombe* with anaphase A contributing relatively little [58,132]. Budding yeast (Figure 6b) is unusual in possessing two members of the kinesin-5 family that cooperate to slide apart antiparallel ipMTs to push apart the spindle pole bodies, with one driving an early rapid phase of spindle elongation and the second driving a slower late phase [32,132]. Ase1p and kinesin-8 also play significant roles in this sliding filament mechanism [117,141], while cytoplasmic aMTs are dispensable for anaphase B [184] and mainly contribute to pulling the spindle/nucleus into the bud neck [185]. Similarly, a cortical pulling mechanism plays very little role in fission yeast anaphase B, which depends virtually exclusively on a midzonal ipMT-MT pushing mechanism [68,71,87], although in this case mediated predominantly by kinesin-6-driven MT-MT sliding, with forces generated by kinesin-5 playing at most a minor role [147]. In both types of yeast, poleward MT flux is thought not to occur and therefore does not contribute to the regulation of anaphase B.

#### 8.2.2. Filamentous and Smut Fungi

Mitosis has been less extensively studied in other divisions of fungi, yet the studies that have been done provide some of the clearest available evidence so far for a cortical pulling mechanism for spindle elongation. For example, laser microbeam studies of living cells of filamentous fungi suggest that outward pulling forces acting on aMTs drive pole–pole separation during anaphase B, with the central spindle serving to constrain the rate of spindle elongation [38]. Subsequently, laser microsurgery and genetic analysis done on the smut fungus, *Ustilago maydis* (Figure 6c), supports the hypothesis that such outward pulling forces are generated by cortical dynein, which drives fast spindle elongation, with kinesin-5 on the midzone driving an initial slow phase of anaphase B [39].

### 8.3. Plants

The mechanism of mitosis in plant cells has long been a topic of great interest [186]. Many plant spindles lack centrosomes (or other discrete MT organizing centers) and aMTs at their poles but, based on EM studies of Haemanthus, they are thought to have “conventional” ipMT bundles constructed from two sets of opposite polarity ipMTs that interdigitate at the midzone and appear to slide apart during anaphase B [24]. Live cell imaging of mitosis in cultured tobacco cells suggests that the spindle elongates, possibly by an ipMT-MT pushing force from about 15 to 20 μm during anaphase B, contributing ≈40% of the final chromosome separation distance [187]. Plant genomes appear to contain large numbers of kinesin and Ase1p family members that could cooperate to drive such a midzonal sliding filament mechanism, e.g., the Arabidopsis genome encodes nine Ase1p MAPs and 61 kinesins, some of which localize to the anaphase spindle midzone, and much effort is aimed at determining their largely unknown functions [188,189]. For example, using fluorescent reporter tagging and live cell imaging in moss, it was found that 43 of 72 kinesins localize to the mitotic spindle and of these almost 30 localized to the antiparallel MT bundles of the anaphase spindle midzone and/or phragmoplast [190] Dissecting the precise functions of these motors, including any roles in anaphase B spindle elongation, represents a daunting yet exciting challenge for plant cell biologists.

### 8.4. Animals

#### 8.4.1. *Caenorhabditis Elegans*

Cortical pulling forces acting on astral MTs represent the major mechanism for exerting outward-directed forces on the spindle poles in *C. elegans* embryos (Figure 6d). These forces, which have been characterized using elegant biophysical experiments [40,100], are thought to underlie both anaphase B spindle elongation and spindle positioning leading to developmentally important asymmetric cell divisions [191,192]. These pulling forces are very plausibly generated by a combination of aMT depolymerization and dynein motors that walk towards the minus ends of aMTs [103,171,191,193], with kinesin-5 at the spindle midzone serving as an antagonistic brake that constrains the rate of pole–pole separation [64]. Interestingly, recent work suggests that a direct interaction between the MT crosslinkers, kinesin-6, and Ase1p/PRC1, could augment the action of kinesin-5 by reinforcing the mechanical resilience of the central spindle to facilitate the dual functions of the midzone during anaphase B and cytokinesis [70]. It is also intriguing that, while cortical pulling represents the dominant mechanism driving spindle elongation in this system, centrosome ablation experiments have revealed the existence of a normally cryptic, redundant mechanism in which ipMT plus end polymerization at the spindle midzone generates an outward force that drives spindle elongation and chromosome segregation during anaphase [108].

Underscoring the diversity of anaphase B mechanisms operating, even within different cells of the same organism, a novel spindle elongation-dependent chromosome segregation mechanism has been found in female meiotic spindles. First, during metaphase, the spindle poles move inward and attach to paired chromosomes; then, during anaphase, the poles are separated carrying the attached homologs with them [109,149]. Since these spindles are anastral, cortical pulling is unlikely, and the authors propose that a motor-driven midzone pushing mechanism drives pole–pole separation, independent of both dynein and kinesin-5 function, but requiring ipMT polymerization. Candidate force generators include one of the aforementioned MT-MT sliding kinesins, a Par-M-type polymerization mechanism and/or an Ase1p-dependent entropic expansion mechanism.

#### 8.4.2. *Drosophila*

Anaphase B spindle elongation in *Drosophila* embryos (Figure 4 and Figure 6e) during cycles 10–13 is thought to depend on a persistent kinesin-5-generated interpolar (ip) microtubule (MT) sliding filament mechanism that “engages” to push apart the spindle poles when poleward flux is turned off [15,60,61]. Based on serial section EM, the spindle poles are linked by “conventional” ipMT bundles whose MTs are crosslinked into a mechanical continuum, possibly by augmin [37,60,194]. Thus, pre-anaphase B spindles are characterized by a force balance in which the outward, kinesin-5-driven sliding of these ipMT bundles is balanced by the kinesin-13-catalyzed depolymerization of their minus ends when they reach the poles, producing poleward flux and maintaining the spindle at a steady length [77,166]. Following cyclin B degradation to initiate anaphase B [180], however, the MT minus-end capping protein, Patronin [163] counteracts kinesin-13 activity at spindle poles to turn off ipMT minus end depolymerization so that poleward flux ceases and the outwardly sliding ipMTs can then elongate the spindle [113]. At the same time, ipMTs display net growth and recruit MT-MT crosslinkers to build a more robust midzone where ipMT-MT sliding forces are generated [62]. Notable among these is Ase1p (aka Feo), which is required for normal spindle elongation as it controls the organization, stability, and motor composition of the midzone, thereby facilitating the kinesin-5-driven sliding filament mechanism underlying proper spindle elongation and chromosome segregation [93].

Here again diversity is found. For example, in cultured S2 cells, the basic mechanism involving an inverse correlation between poleward flux and spindle elongation is observed, but quantitative differences exist, for example flux is only partially turned off at anaphase B onset leading to more variance in rates of spindle elongation [63]. Moreover, mitotic spindles in extracts prepared from embryos during cycles 6–7 lack cortices but appear to utilize cytoplasmic astral pulling forces during anaphase spindle elongation [104]. The significance of these differences is unclear.

#### 8.4.3. Vertebrates

The observation using EM that the minus ends of ipMT bundles in vertebrate-cultured cells do not reach the poles suggests that they do not directly push the poles apart [90], even though ipMTs slide apart and polymerize at the midzone as the anaphase B spindle elongates [33], plausibly assisted by the augmin-/γ-TuRC-dependent branching polymerization of ipMT plus ends [107] (Figure 6f). Various studies support the idea that forces exerted at the cortex, presumably by dynein and/or aMT depolymerization, pull the poles apart while these forces are resisted by antagonistic forces generated at the midzone [66,173]. Moreover, inhibitors that weaken or enhance MT binding by kinesin-5 lead to an increased or decreased rate of spindle elongation, respectively, suggesting that midzonal kinesin-5 acts as a brake that restricts anaphase B [65]. These studies were done mainly on mammalian cultured cells, and of course may not be applicable to all vertebrate cells, where it is well established that great diversity exists e.g., anaphase B is unusual in preceding anaphase A in mouse eggs [42]. It is also worth noting that *Xenopus* extract spindles, which have been so influential in studies of many aspects of the mechanism of mitosis [110,195,196], have contributed less to our understanding of anaphase B spindle elongation, which is not a robust feature of extract spindles, possibly because of the absence of cortices [197].

### 8.5. Prokaryotes

The R1 plasmid segregation system of *E. coli* represents a striking example of an anaphase B-like DNA segregation system operating in bacteria (Figure 6g). A large body of work suggests that an antiparallel bundle of ParM filaments, with their plus-ends facing outward and attached to ParR-coated centromeric DNA, can polymerize and exert force by a polymer ratchet mechanism to push pairs of sister plasmids to opposite ends of the cell [10,174,175,176]. Thus the bidirectional polymerization, rather than the sliding apart, of bundles of antiparallel cytoskeletal polymers drives spindle elongation.

## 9. Theoretical Models of Anaphase B

**Figure 7:**
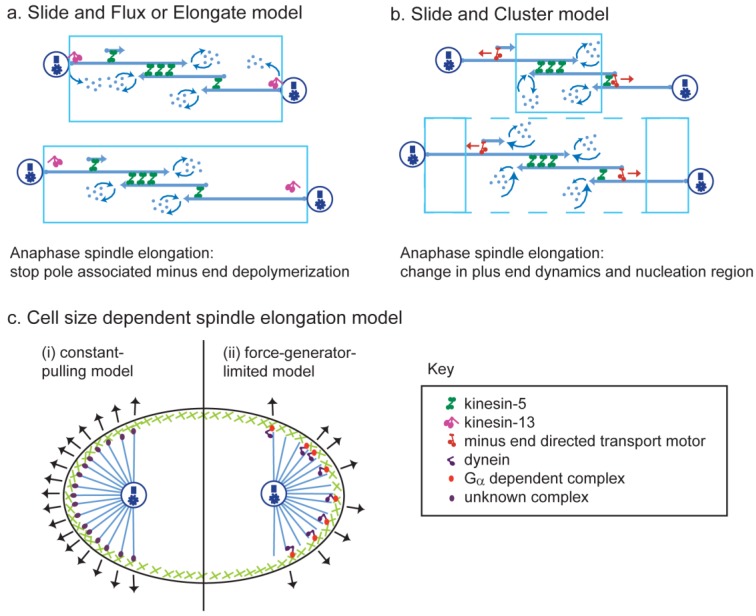
Theoretical models of anaphase B. Three of the models discussed in the text are drawn here, namely (**a**) the slide and flux-or-elongate (SAFE) model; (**b**) the slide-and-cluster (SAC) model; and (**c**) the cell-size dependent spindle elongation model. ((**a**) and (**b**) are based on [199] and (**c**) is based on [192].)

Because of the molecular complexity of the machinery that mediates anaphase B spindle elongation, it is appreciated that a full understanding of the molecular mechanism of anaphase B will require theoretical/quantitative modeling [198]. Theoretical models (Figure 7) that incorporate realistic properties of the spindle and its molecular components can yield solutions that illuminate e.g., the factors that govern the mechanical design of the elongating spindle, describe its dynamic evolution, in plots of spindle length versus time, and display spindle dynamics in a visually accessible manner using computer simulations. Model solutions can also illuminate features of anaphase B that cannot be revealed through intuition and, most useful of all, yield predictions that can be tested experimentally.

An early example of a theoretical model that has strongly influenced our thinking about the mechanism of anaphase B spindle elongation was the sliding filament model [22]. This qualitative model was initially proposed in order to explain the mechanism of mitosis, including both anaphase A and B, based on the sliding apart of adjacent, structurally polar spindle MTs driven by ATP-hydrolyzing mechanochemical cross-bridges (aka mitotic motors). The model was useful because it made precise and unique predictions about the relative polarity patterns of the spindle MTs, leading to the development of methods for carefully testing these predictions by EM (see above). The results essentially ruled out the sliding filament mechanism for chromosome-to-pole motion during anaphase A, which is now understood to depend upon some kind of “pacman-flux” mechanism associated with the shortening of kMTs, but they do strongly support an outward ipMT sliding filament mechanism for anaphase B spindle elongation.

A different type of theoretical approach illuminates the mechanical design principles of the “beam-like” fission yeast mitotic spindle undergoing anaphase B spindle elongation [88]. These spindles use an antiparallel ipMT-MT sliding filament mechanism generated at the midzone to elongate against opposing compressive forces that could potentially cause spindle buckling. Theoretical considerations of the physical principles operating in these spindles, combined with computer simulations and structural analysis via EM tomography, led the authors to propose that the compressive strength of the elongating spindles is optimized to support the drag forces that resist spindle elongation. This is accomplished by crosslinking the ipMTs into rigid, paracrystalline arrays that display square and hexagonal symmetry within and outside the central midzone, respectively, and by preserving the optimal number and net length of the ipMTs.

A “slide and flux-or-elongate” (SAFE) model based on spindle geometry and the biochemical properties of spindle components was used to model the dynamics of the transition between the pre-anaphase B (i.e., metaphase/anaphase A) spindle that maintains a constant steady state length and anaphase B spindle elongation in *Drosophila* embryos [60] (Figure 7a). Where possible, this force balance model was guided by experimental data on e.g., the geometry of embryo spindles including the three-dimensional organization and dynamic properties of MTs within ipMT bundles and the force velocity properties of mitotic motors. These properties were incorporated into a set of differential equations that describe the kinematics of the spindle poles, the kinematics of the overlapping ipMTs at the spindle midzone, and the ipMT-generated forces acting on the spindle poles. The solution of these model equations faithfully recapitulates spindle dynamics throughout the pre-anaphase B steady state, when the plus ends of the overlapping ipMTs at the midzone are slid apart and depolymerize at their minus ends, giving rise to poleward ipMT flux and anaphase B spindle elongation, which is initiated by the cessation of ipMT depolymerization at the poles, turning off flux and allowing the sliding ipMTs to push apart the poles. The model solution describes how bipolar kinesin-5 motors slide apart the dynamically unstable ipMTs at the midzone to produce the steady, linear rate of elongation that is observed in vivo and showed that the elongation rate is determined solely by the rate of ipMT sliding combined with the extent of suppression of ipMT depolymerization at the poles. It also made interesting, experimentally testable predictions, e.g., that the rate of elongation was robust to substantial changes in the number of sliding motors and ipMTs and to the dynamic instability properties of the ipMTs, but it suggested that the plus ends of these dynamic ipMTs must undergo net polymerization in order to sustain the robust, linear spindle elongation observed. Models superficially similar to this model have also been proposed to account for spindle length control in *Xenopus* extracts, where consistent anaphase B is not observed [200] and in cultured human cells, where an Aurora B gradient regulates the minus end depolymerization, length, and alignment of ipMTs that slide apart at their plus ends to determine the length of the central spindle that forms during late anaphase [201].

A very different force balance model, the “slide and cluster (SAC) model” [110] (Figure 7b), somewhat surprisingly, can also account for important features of anaphase B spindle elongation. In this model, which was initially developed to account for the control of metaphase meiotic spindle steady state length in *Xenopus* extracts, antiparallel MTs are nucleated around chromatin at the spindle midzone and are slid outward by kinesin-5. Around the equator, a minus-end-directed motor assists kinesin-5 by transporting the nucleated MTs, minus ends leading, along parallel MT tracks towards the spindle poles where it then opposes kinesin-5 and clusters the transported MTs to focus the poles. In this model, spindle length is determined by the rate of transport of the poleward sliding MTs combined with their lifetime, which in turn depends on the dynamic instability properties of their plus (not minus) ends. Thus, it was postulated, for example, that a change in plus end dynamics of these MTs, e.g., a decrease in their catastrophe frequency, could induce spindle elongation to a new steady state length, mimicking anaphase B and this idea was recently tested [199]. Despite the considerable differences in architecture that exist between *Xenopus* extract and *Drosophila* embryo spindles, quantitative computational modeling suggests that the SAC model can explain many, but not all, aspects of anaphase B spindle dynamics in *Drosophila* embryos almost as well as the SAFE model can. It was thus concluded that the SAFE model provides a more realistic description of the underlying molecular mechanism of anaphase B spindle elongation, at least in *Drosophila* embryos.

A quantitative model has been proposed to describe cell size-dependent anaphase B spindle elongation in *C. elegans* early embryos, invoking cortical force generators acting on astral MTs to pull apart the spindle poles [192] (Figure 7c). In this system, quantitative measurements revealed that the rate and extent of anaphase B spindle elongation, which govern the post-anaphase B spindle length, correlate with cell size. Two models for the cortical pulling mechanism controlling anaphase B spindle elongation were considered: (i) the “constant pulling model” for heterotrimeric G-protein (Gα)-independent spindle elongation in which astral MTs are pulled outward with a constant force; and (ii) the “force generator-limited model” for Gα-dependent spindle elongation, in which the density of force generators per unit area of cortex is constant and independent of cell size. In computer simulations, these two models could account for the observed dynamics of anaphase B spindle elongation that are seen in vivo. Specifically, simulations of the first model reproduced only the cell-size dependency of the extent of spindle elongation but not its speed, which remained constant with cell size, a situation that agrees with observations of Gα-disrupted cells. However, simulations using a combination of the first and second models reproduced observations in wild-type embryonic spindles, in which the extent and the speed of spindle elongation, as well as the resulting post-anaphase B spindle length, are all cell size-dependent.

## 10. Concluding Remarks

Anaphase B spindle elongation represents arguably one of the simplest sub-routines in the mechanism of mitosis, yet an elaborate and diverse machinery has evolved to accomplish pole–pole separation over distances of a few microns in the various systems that have so far been studied. Indeed, more variety than we currently appreciate may exist as more exotic mitotic mechanisms are uncovered in different cells and organisms [183], a view supported by the striking discovery that *C. elegans* meiotic spindles utilize a novel anaphase B mechanism to segregate chromosomes [149]. We have found it useful to describe the currently known mechanisms of anaphase B in terms of the differential deployment of a few conserved biochemical modules in different cell types, i.e., midzone pushing and braking via ipMT-MT crosslinking; cortical pulling, ipMT plus end dynamics/net polymerization, and minus end depolymerization/poleward flux. One aim of the research on this topic is to elucidate the molecular mechanism of these fundamental processes in atomic detail at high temporal resolution, where progress is being made, e.g., [125,128,175,202]. Progress is also being made in the reconstitution of some of these basic processes from purified components, which represents a powerful direction for investigating their underlying molecular mechanisms [154,176]. Although not a major focus of the current review, the regulation of anaphase B represents an important area for future studies. Current evidence suggests that this may be a very complex and dynamic process, making mathematical modeling an essential tool for understanding the mechanisms at work [177]. In addition, given the elaborate and diverse nature of the cytoskeleton-based mechanisms that mediate anaphase B and other aspects of mitosis and cell division among present-day eukaryotic and bacterial cells, a fascinating unknown is how these machineries and mechanisms originated and evolved from the purely physical mechanisms that were thought to operate in dividing ancestral protocells on the early Earth [203].

## Figures and Tables

**Table 1:** Rates and extent of spindle length changes during anaphase B.

Organism	Rate of Spindle Elongation	Extent of Spindle Elongation from Metaphase to Telophase	Reference(s)
Diatom	Live: 0.038 ± 0.005 μm/s	~2 μm	[45]
	Isolated: 0.015 ± 0.002 μm/s *	1.9 ± 0.17 μm	[44]
*Ustilago maydis*	Slow: 0.02 ± 0.003 μm/s	~0.5 μm	[39]
	Fast: 0.09 ± 0.003 μm/s	~4.5 μm	
*Schizosaccharomyces pombe*	~0.013 μm/s	7–10 μm	[58]
*Saccharomyces cerevisiae*	Fast: 0.018 ± 0.005 μm/s	~4 μm	[47]
	Slow: ~0.006 μm/s	2–6 μm	
*Drosophila* syncytial embryo	0.08 ± 0.015 μm/s	5 μm	[61]
	(cycle 12)		
S2 cell	0.017 μm/s	5 μm	[63]
*C. elegans*	0.107 ± 0.008 μm/s	8.33 ± 0.29 μm	[64]
LLC-Pk1 epithelial cells	0.049 ± 0.017 μm/s	8.73 ± 2.4 μm	[65]

* varies depending on assay conditions.

## References

[1] McIntosh J.R., Molodtsov M.I., Ataullakhanov F.I. (2012). Biophysics of mitosis. Q. Rev. Biophys..

[2] Walczak C.E., Cai S., Khodjakov A. (2010). Mechanisms of chromosome behaviour during mitosis. Nat. Rev. Mol. Cell Biol..

[3] Goshima G., Scholey J.M. (2010). Control of mitotic spindle length. Annu. Rev. Cell Dev. Biol..

[4] Maiato H., Lince-Faria M. (2010). The perpetual movements of anaphase. Cell Mol. Life Sci..

[5] Gadde S., Heald R. (2004). Mechanisms and molecules of the mitotic spindle. Curr. Biol..

[6] Cross R.A., McAinsh A. (2014). Prime movers: The mechanochemistry of mitotic kinesins. Nat. Rev. Mol. Cell Biol..

[7] Petry S. (2016). Mechanisms of mitotic spindle assembly. Annu. Rev. Biochem..

[8] Bouck D.C., Joglekar A.P., Bloom K.S. (2008). Design features of a mitotic spindle: Balancing tension and compression at a single microtubule kinetochore interface in budding yeast. Annu. Rev. Genet..

[9] Maddox P.S., Oegema K., Desai A., Cheeseman I.M. (2004). “Holo”er than thou: Chromosome segregation and kinetochore function in *C. elegans*. Chromosome Res..

[10] Gayathri P., Fujii T., Moller-Jensen J., van den Ent F., Namba K., Lowe J. (2012). A bipolar spindle of antiparallel ParM filaments drives bacterial plasmid segregation. Science.

[11] Cimini D., Cameron L.A., Salmon E.D. (2004). Anaphase spindle mechanics prevent mis-segregation of merotelically oriented chromosomes. Curr. Biol..

[12] Courtheoux T., Gay G., Gachet Y., Tournier S. (2009). Ase1/PRC1-dependent spindle elongation corrects merotely during anaphase in fission yeast. J. Cell Biol..

[13] Pidoux A.L., Uzawa S., Perry P.E., Cande W.Z., Allshire R.C. (2000). Live analysis of lagging chromosomes during anaphase and their effect on spindle elongation rate in fission yeast. J. Cell Sci..

[14] Ford J.H. (2013). Protraction of anaphase B in lymphocyte mitosis with ageing: Possible contribution to age-related cancer risk. Mutagenesis.

[15] Brust-Mascher I., Scholey J.M. (2011). Mitotic motors and chromosome segregation: The mechanism of anaphase B. Biochem. Soc. Trans..

[16] Cande W.Z., Hogan C.J. (1989). The mechanism of anaphase spindle elongation. Bioessays.

[17] Roostalu J., Schiebel E., Khmelinskii A. (2010). Cell cycle control of spindle elongation. Cell Cycle.

[18] Ris H. (1943). A quantitative study of anaphase movement in the aphid tamalia. Biol. Bull..

[19] Ris H. (1949). The anaphase movement of chromosomes in the spermatocytes of the grasshopper. Biol. Bull..

[20] Rapaport R. (1996). Cytokinesis in Animal Cells.

[21] Schrader F. (1949). Mitosis. The Movement of Chromosomes during Cell Division.

[22] McIntosh J.R., Hepler P.K., van Wie D.G. (1969). Model for mitosis. Nature.

[23] Huxley H., Hanson J. (1954). Changes in the cross-striations of muscle during contraction and stretch and their structural interpretation. Nature.

[24] Euteneuer U., Jackson W.T., McIntosh J.R. (1982). Polarity of spindle microtubules in haemanthus endosperm. J. Cell Biol..

[25] Hepler P.K., McIntosh J.R., Cleland S. (1970). Intermicrotubule bridges in mitotic spindle apparatus. J. Cell Biol..

[26] McDonald K., Pickett-Heaps J.D., McIntosh J.R., Tippit D.H. (1977). On the mechanism of anaphase spindle elongation in diatoma vulgare. J. Cell Biol..

[27] McIntosh J.R., Landis S.C. (1971). The distribution of spindle microtubules during mitosis in cultured human cells. J. Cell Biol..

[28] Kashina A.S., Baskin R.J., Cole D.G., Wedaman K.P., Saxton W.M., Scholey J.M. (1996). A bipolar kinesin. Nature.

[29] Scholey J.M., Porter M.E., Grissom P.M., McIntosh J.R. (1985). Identification of kinesin in sea urchin eggs, and evidence for its localization in the mitotic spindle. Nature.

[30] Kapitein L.C., Peterman E.J., Kwok B.H., Kim J.H., Kapoor T.M., Schmidt C.F. (2005). The bipolar mitotic kinesin Eg5 moves on both microtubules that it crosslinks. Nature.

[31] Cole D.G., Saxton W.M., Sheehan K.B., Scholey J.M. (1994). A “slow” homotetrameric kinesin-related motor protein purified from *Drosophila* embryos. J. Biol. Chem..

[32] Winey M., Mamay C.L., O’Toole E.T., Mastronarde D.N., Giddings T.H., McDonald K.L., McIntosh J.R. (1995). Three-dimensional ultrastructural analysis of the *Saccharomyces cerevisiae* mitotic spindle. J. Cell Biol..

[33] Saxton W.M., McIntosh J.R. (1987). Interzone microtubule behavior in late anaphase and telophase spindles. J. Cell Biol..

[34] Cande W.Z., McDonald K. (1986). Physiological and ultrastructural analysis of elongating mitotic spindles reactivated in vitro. J. Cell Biol..

[35] Cande W.Z., McDonald K.L. (1985). In vitro reactivation of anaphase spindle elongation using isolated diatom spindles. Nature.

[36] Hogan C.J., Wein H., Wordeman L., Scholey J.M., Sawin K.E., Cande W.Z. (1993). Inhibition of anaphase spindle elongation in vitro by a peptide antibody that recognizes kinesin motor domain. Proc. Natl. Acad. Sci. USA.

[37] Sharp D.J., McDonald K.L., Brown H.M., Matthies H.J., Walczak C., Vale R.D., Mitchison T.J., Scholey J.M. (1999). The bipolar kinesin, KLP61F, cross-links microtubules within interpolar microtubule bundles of *Drosophila* embryonic mitotic spindles. J. Cell Biol..

[38] Aist J.R., Bayles C.J., Tao W., Berns M.W. (1991). Direct experimental evidence for the existence, structural basis and function of astral forces during anaphase B in vivo. J. Cell Sci..

[39] Fink G., Schuchardt I., Colombelli J., Stelzer E., Steinberg G. (2006). Dynein-mediated pulling forces drive rapid mitotic spindle elongation in *ustilago maydis*. EMBO J..

[40] Grill S.W., Gonczy P., Stelzer E.H., Hyman A.A. (2001). Polarity controls forces governing asymmetric spindle positioning in the *Caenorhabditis elegans* embryo. Nature.

[41] Waters J.C., Cole R.W., Rieder C.L. (1993). The force-producing mechanism for centrosome separation during spindle formation in vertebrates is intrinsic to each aster. J. Cell Biol..

[42] FitzHarris G. (2012). Anaphase B precedes anaphase A in the mouse egg. Curr. Biol..

[43] Yanagida M. (2014). The role of model organisms in the history of mitosis research. Cold Spring Harb. Perspect. Biol..

[44] Baskin T.I., Cande W.Z. (1990). Kinetic analysis of mitotic spindle elongation in vitro. J. Cell Sci..

[45] Cohn S.A., Pickett-Heaps J.D. (1988). The effects of colchicine and dinitrophenol on the in vivo rates of anaphase A and B in the diatom *surirella*. Eur. J. Cell Biol..

[46] Brust-Mascher I., Scholey J.M. (2007). Mitotic spindle dynamics in *Drosophila*. Int. Rev. Cytol..

[47] Yeh E., Skibbens R.V., Cheng J.W., Salmon E.D., Bloom K. (1995). Spindle dynamics and cell cycle regulation of dynein in the budding yeast, *Saccharomyces cerevisiae*. J. Cell Biol..

[48] Desai A., Mitchison T.J. (1997). Microtubule polymerization dynamics. Annu. Rev. Cell Dev. Biol..

[49] Inoue S., Salmon E.D. (1995). Force generation by microtubule assembly/disassembly in mitosis and related movements. Mol. Biol. Cell.

[50] Rogers G.C., Rogers S.L., Sharp D.J. (2005). Spindle microtubules in flux. J. Cell Sci..

[51] Salmon E.D., Leslie R.J., Saxton W.M., Karow M.L., McIntosh J.R. (1984). Spindle microtubule dynamics in sea urchin embryos: Analysis using a fluorescein-labeled tubulin and measurements of fluorescence redistribution after laser photobleaching. J. Cell Biol..

[52] Zhai Y., Kronebusch P.J., Simon P.M., Borisy G.G. (1996). Microtubule dynamics at the g2/m transition: Abrupt breakdown of cytoplasmic microtubules at nuclear envelope breakdown and implications for spindle morphogenesis. J. Cell Biol..

[53] Mitchison T.J. (1989). Polewards microtubule flux in the mitotic spindle: Evidence from photoactivation of fluorescence. J. Cell Biol..

[54] Mitchison T.J., Salmon E.D. (1992). Poleward kinetochore fiber movement occurs during both metaphase and anaphase-A in newt lung cell mitosis. J. Cell Biol..

[55] Waterman-Storer C.M., Salmon E.D. (1999). Fluorescent speckle microscopy of microtubules: How low can you go?. FASEB J..

[56] Higuchi T., Uhlmann F. (2005). Stabilization of microtubule dynamics at anaphase onset promotes chromosome segregation. Nature.

[57] Maddox P.S., Bloom K.S., Salmon E.D. (2000). The polarity and dynamics of microtubule assembly in the budding yeast *Saccharomyces cerevisiae*. Nat. Cell Biol..

[58] Mallavarapu A., Sawin K., Mitchison T. (1999). A switch in microtubule dynamics at the onset of anaphase B in the mitotic spindle of *Schizosaccharomyces pombe*. Curr. Biol..

[59] Zhai Y., Kronebusch P.J., Borisy G.G. (1995). Kinetochore microtubule dynamics and the metaphase-anaphase transition. J. Cell Biol..

[60] Brust-Mascher I., Civelekoglu-Scholey G., Kwon M., Mogilner A., Scholey J.M. (2004). Model for anaphase B: Role of three mitotic motors in a switch from poleward flux to spindle elongation. Proc. Natl. Acad. Sci. USA.

[61] Brust-Mascher I., Scholey J.M. (2002). Microtubule flux and sliding in mitotic spindles of *Drosophila* embryos. Mol. Biol. Cell.

[62] Cheerambathur D.K., Civelekoglu-Scholey G., Brust-Mascher I., Sommi P., Mogilner A., Scholey J.M. (2007). Quantitative analysis of an anaphase B switch: Predicted role for a microtubule catastrophe gradient. J. Cell Biol..

[63] De Lartigue J., Brust-Mascher I., Scholey J.M. (2011). Anaphase B spindle dynamics in *Drosophila* s2 cells: Comparison with embryo spindles. Cell Div..

[64] Saunders A.M., Powers J., Strome S., Saxton W.M. (2007). Kinesin-5 acts as a brake in anaphase spindle elongation. Curr. Biol..

[65] Collins E., Mann B.J., Wadsworth P. (2014). Eg5 restricts anaphase B spindle elongation in mammalian cells. Cytoskeleton (Hoboken).

[66] Aist J.R., Liang H., Berns M.W. (1993). Astral and spindle forces in PTK2 cells during anaphase B: A laser microbeam study. J. Cell Sci..

[67] Leslie R.J., Pickett-Heaps J.D. (1983). Ultraviolet microbeam irradiations of mitotic diatoms: Investigation of spindle elongation. J. Cell Biol..

[68] Khodjakov A., La Terra S., Chang F. (2004). Laser microsurgery in fission yeast; role of the mitotic spindle midzone in anaphase B. Curr. Biol..

[69] Verbrugghe K.J., White J.G. (2004). SPD-1 is required for the formation of the spindle midzone but is not essential for the completion of cytokinesis in *C. elegans* embryos. Curr. Biol..

[70] Lee K.Y., Esmaeili B., Zealley B., Mishima M. (2015). Direct interaction between centralspindlin and PRC1 reinforces mechanical resilience of the central spindle. Nat. Commun..

[71] Tolic-Norrelykke I.M., Sacconi L., Thon G., Pavone F.S. (2004). Positioning and elongation of the fission yeast spindle by microtubule-based pushing. Curr. Biol..

[72] Nicklas R.B. (1965). Chromosome velocity during mitosis as a function of chromosome size and position. J. Cell Biol..

[73] Hiramoto Y. (1969). Mechanical properties of the protoplasm of the sea urchin egg. II. Fertilized egg. Exp. Cell Res..

[74] Scholey J.M. (2013). Compare and contrast the reaction coordinate diagrams for chemical reactions and cytoskeletal force generators. Mol. Biol. Cell.

[75] Nicklas R.B. (1983). Measurements of the force produced by the mitotic spindle in anaphase. J. Cell Biol..

[76] Hiramoto Y., Nakano Y. (1988). Micromanipulation studies of the mitotic apparatus in sand dollar eggs. Cell Motil. Cytoskelet..

[77] Brust-Mascher I., Sommi P., Cheerambathur D.K., Scholey J.M. (2009). Kinesin-5-dependent poleward flux and spindle length control in *Drosophila* embryo mitosis. Mol. Biol. Cell.

[78] Nicklas R.B. (1984). A quantitative comparison of cellular motile systems. Cell Motil..

[79] Cande W.Z. (1982). Nucleotide requirements for anaphase chromosome movements in permeabilized mitotic cells: Anaphase B but not anaphase A requires ATP. Cell.

[80] Cande W.Z. (1983). Creatine kinase role in anaphase chromosome movement. Nature.

[81] Koons S.J., Eckert B.S., Zobel C.R. (1982). Immunofluorescence and inhibitor studies on creatine kinase and mitosis. Exp. Cell Res..

[82] Palazzo R.E., Lutz D.A., Rebhun L.I. (1991). Reactivation of isolated mitotic apparatus: Metaphase versus anaphase spindles. Cell Motil. Cytoskelet..

[83] Civelekoglu-Scholey G., Scholey J.M. (2010). Mitotic force generators and chromosome segregation. Cell Mol. Life Sci..

[84] Janson M.E., Loughlin R., Loiodice I., Fu C., Brunner D., Nedelec F.J., Tran P.T. (2007). Crosslinkers and motors organize dynamic microtubules to form stable bipolar arrays in fission yeast. Cell.

[85] Lansky Z., Braun M., Ludecke A., Schlierf M., ten Wolde P.R., Janson M.E., Diez S. (2015). Diffusible crosslinkers generate directed forces in microtubule networks. Cell.

[86] Heidemann S.R., McIntosh J.R. (1980). Visualization of the structural polarity of microtubules. Nature.

[87] Ding R., McDonald K.L., McIntosh J.R. (1993). Three-dimensional reconstruction and analysis of mitotic spindles from the yeast, *Schizosaccharomyces pombe*. J. Cell Biol..

[88] Ward J.J., Roque H., Antony C., Nedelec F. (2014). Mechanical design principles of a mitotic spindle. Elife.

[89] Huxley H.E. (1963). Electron microscope studies on the structure of natural and synthetic protein filaments from striated muscle. J. Mol. Biol..

[90] Mastronarde D.N., McDonald K.L., Ding R., McIntosh J.R. (1993). Interpolar spindle microtubules in PTK cells. J. Cell Biol..

[91] Wilson H.J. (1969). Arms and bridges on microtubules in the mitotic apparatus. J. Cell Biol..

[92] Cheerambathur D.K., Brust-Mascher I., Civelekoglu-Scholey G., Scholey J.M. (2008). Dynamic partitioning of mitotic kinesin-5 cross-linkers between microtubule-bound and freely diffusing states. J. Cell Biol..

[93] Wang H., Brust-Mascher I., Scholey J.M. (2015). The microtubule cross-linker Feo controls the midzone stability, motor composition, and elongation of the anaphase B spindle in *Drosophila* embryos. Mol. Biol. Cell.

[94] Acar S., Carlson D.B., Budamagunta M.S., Yarov-Yarovoy V., Correia J.J., Ninonuevo M.R., Jia W., Tao L., Leary J.A., Voss J.C. (2013). The bipolar assembly domain of the mitotic motor kinesin-5. Nat. Commun..

[95] Glotzer M. (2009). The 3ms of central spindle assembly: Microtubules, motors and maps. Nat. Rev. Mol. Cell Biol..

[96] Peterman E.J., Scholey J.M. (2009). Mitotic microtubule crosslinkers: Insights from mechanistic studies. Curr. Biol..

[97] Rozelle D.K., Hansen S.D., Kaplan K.B. (2011). Chromosome passenger complexes control anaphase duration and spindle elongation via a kinesin-5 brake. J. Cell Biol..

[98] Shimamoto Y., Forth S., Kapoor T.M. (2015). Measuring pushing and braking forces generated by ensembles of kinesin-5 crosslinking two microtubules. Dev. Cell.

[99] Tikhonenko I., Nag D.K., Martin N., Koonce M.P. (2008). Kinesin-5 is not essential for mitotic spindle elongation in *Dictyostelium*. Cell Motil. Cytoskelet..

[100] Grill S.W., Howard J., Schaffer E., Stelzer E.H., Hyman A.A. (2003). The distribution of active force generators controls mitotic spindle position. Science.

[101] Grill S.W., Hyman A.A. (2005). Spindle positioning by cortical pulling forces. Dev. Cell.

[102] Grishchuk E.L., Molodtsov M.I., Ataullakhanov F.I., McIntosh J.R. (2005). Force production by disassembling microtubules. Nature.

[103] Kozlowski C., Srayko M., Nedelec F. (2007). Cortical microtubule contacts position the spindle in *C. elegans* embryos. Cell.

[104] Telley I.A., Gaspar I., Ephrussi A., Surrey T. (2012). Aster migration determines the length scale of nuclear separation in the *Drosophila* syncytial embryo. J. Cell Biol..

[105] Masuda H., Cande W.Z. (1987). The role of tubulin polymerization during spindle elongation in vitro. Cell.

[106] Masuda H., McDonald K.L., Cande W.Z. (1988). The mechanism of anaphase spindle elongation: Uncoupling of tubulin incorporation and microtubule sliding during in vitro spindle reactivation. J. Cell Biol..

[107] Uehara R., Goshima G. (2010). Functional central spindle assembly requires de novo microtubule generation in the interchromosomal region during anaphase. J. Cell Biol..

[108] Nahaboo W., Zouak M., Askjaer P., Delattre M. (2015). Chromatids segregate without centrosomes during *Caenorhabditis elegans* mitosis in a ran- and clasp-dependent manner. Mol. Biol. Cell.

[109] Dumont J., Oegema K., Desai A. (2010). A kinetochore-independent mechanism drives anaphase chromosome separation during acentrosomal meiosis. Nat. Cell Biol..

[110] Burbank K.S., Mitchison T.J., Fisher D.S. (2007). Slide-and-cluster models for spindle assembly. Curr. Biol..

[111] Hays T.S., Wise D., Salmon E.D. (1982). Traction force on a kinetochore at metaphase acts as a linear function of kinetochore fiber length. J. Cell Biol..

[112] Ostergren G. (1950). Considerations on some elementary features of mitosis. Hereditas.

[113] Wang H., Brust-Mascher I., Civelekoglu-Scholey G., Scholey J.M. (2013). Patronin mediates a switch from kinesin-13-dependent poleward flux to anaphase B spindle elongation. J. Cell Biol..

[114] Akhmanova A., Steinmetz M.O. (2015). Control of microtubule organization and dynamics: Two ends in the limelight. Nat. Rev. Mol. Cell Biol..

[115] Goldstein L.S. (1993). Functional redundancy in mitotic force generation. J. Cell Biol..

[116] Pellman D., Bagget M., Tu Y.H., Fink G.R., Tu H. (1995). Two microtubule-associated proteins required for anaphase spindle movement in saccharomyces cerevisiae. J. Cell Biol..

[117] Schuyler S.C., Liu J.Y., Pellman D. (2003). The molecular function of Ase1p: Evidence for a map-dependent midzone-specific spindle matrix. Microtubule-associated proteins. J. Cell Biol..

[118] Kapitein L.C., Janson M.E., van den Wildenberg S.M., Hoogenraad C.C., Schmidt C.F., Peterman E.J. (2008). Microtubule-driven multimerization recruits Ase1p onto overlapping microtubules. Curr. Biol..

[119] Subramanian R., Ti S.C., Tan L., Darst S.A., Kapoor T.M. (2013). Marking and measuring single microtubules by PRC1 and kinesin-4. Cell.

[120] Khmelinskii A., Roostalu J., Roque H., Antony C., Schiebel E. (2009). Phosphorylation-dependent protein interactions at the spindle midzone mediate cell cycle regulation of spindle elongation. Dev. Cell.

[121] Wein H., Bass H.W., Cande W.Z. (1998). Dsk1, a kinesin-related protein involved in anaphase spindle elongation, is a component of a mitotic spindle matrix. Cell Motil. Cytoskelet..

[122] Odde D.J. (2015). Mitosis, diffusible crosslinkers, and the ideal gas law. Cell.

[123] Jiang H., Wang S., Huang Y., He X., Cui H., Zhu X., Zheng Y. (2015). Phase transition of spindle-associated protein regulate spindle apparatus assembly. Cell.

[124] Civelekoglu-Scholey G., Tao L., Brust-Mascher I., Wollman R., Scholey J.M. (2010). Prometaphase spindle maintenance by an antagonistic motor-dependent force balance made robust by a disassembling lamin-b envelope. J. Cell Biol..

[125] Scholey J.E., Nithianantham S., Scholey J.M., Al-Bassam J. (2014). Structural basis for the assembly of the mitotic motor kinesin-5 into bipolar tetramers. Elife.

[126] Sawin K.E., Le Guellec K., Philippe M., Mitchison T.J. (1992). Mitotic spindle organization by a plus-end-directed microtubule motor. Nature.

[127] Valentine M.T., Gilbert S.P. (2007). To step or not to step? How biochemistry and mechanics influence processivity in kinesin and Eg5. Curr. Opin. Cell Biol..

[128] Valentine M.T., Fordyce P.M., Krzysiak T.C., Gilbert S.P., Block S.M. (2006). Individual dimers of the mitotic kinesin motor Eg5 step processively and support substantial loads in vitro. Nat. Cell Biol..

[129] Hildebrandt E.R., Gheber L., Kingsbury T., Hoyt M.A. (2006). Homotetrameric form of cin8p, a *Saccharomyces cerevisiae* kinesin-5 motor, is essential for its in vivo function. J. Biol. Chem..

[130] Van den Wildenberg S.M., Tao L., Kapitein L.C., Schmidt C.F., Scholey J.M., Peterman E.J. (2008). The homotetrameric kinesin-5 KLP61F preferentially crosslinks microtubules into antiparallel orientations. Curr. Biol..

[131] Enos A.P., Morris N.R. (1990). Mutation of a gene that encodes a kinesin-like protein blocks nuclear division in *A. nidulans*. Cell.

[132] Straight A.F., Sedat J.W., Murray A.W. (1998). Time-lapse microscopy reveals unique roles for kinesins during anaphase in budding yeast. J. Cell Biol..

[133] Saunders W.S., Koshland D., Eshel D., Gibbons I.R., Hoyt M.A. (1995). *Saccharomyces cerevisiae* kinesin- and dynein-related proteins required for anaphase chromosome segregation. J. Cell Biol..

[134] Avunie-Masala R., Movshovich N., Nissenkorn Y., Gerson-Gurwitz A., Fridman V., Koivomagi M., Loog M., Hoyt M.A., Zaritsky A., Gheber L. (2011). Phospho-regulation of kinesin-5 during anaphase spindle elongation. J. Cell Sci..

[135] Sharp D.J., Brown H.M., Kwon M., Rogers G.C., Holland G., Scholey J.M. (2000). Functional coordination of three mitotic motors in *Drosophila* embryos. Mol. Biol. Cell.

[136] Roostalu J., Hentrich C., Bieling P., Telley I.A., Schiebel E., Surrey T. (2011). Directional switching of the kinesin cin8 through motor coupling. Science.

[137] Gerson-Gurwitz A., Thiede C., Movshovich N., Fridman V., Podolskaya M., Danieli T., Lakamper S., Klopfenstein D.R., Schmidt C.F., Gheber L. (2011). Directionality of individual kinesin-5 cin8 motors is modulated by loop 8, ionic strength and microtubule geometry. EMBO J..

[138] Edamatsu M. (2014). Bidirectional motility of the fission yeast kinesin-5, cut7. Biochem. Biophys. Res. Commun..

[139] Kwon M., Morales-Mulia S., Brust-Mascher I., Rogers G.C., Sharp D.J., Scholey J.M. (2004). The chromokinesin, KLP3a, dives mitotic spindle pole separation during prometaphase and anaphase and facilitates chromatid motility. Mol. Biol. Cell.

[140] Nislow C., Lombillo V.A., Kuriyama R., McIntosh J.R. (1992). A plus-end-directed motor enzyme that moves antiparallel microtubules in vitro localizes to the interzone of mitotic spindles. Nature.

[141] Su X., Arellano-Santoyo H., Portran D., Gaillard J., Vantard M., Thery M., Pellman D. (2013). Microtubule-sliding activity of a kinesin-8 promotes spindle assembly and spindle-length control. Nat. Cell Biol..

[142] Varga V., Leduc C., Bormuth V., Diez S., Howard J. (2009). Kinesin-8 motors act cooperatively to mediate length-dependent microtubule depolymerization. Cell.

[143] Rizk R.S., Discipio K.A., Proudfoot K.G., Gupta M.L. (2014). The kinesin-8 kip3 scales anaphase spindle length by suppression of midzone microtubule polymerization. J. Cell Biol..

[144] Mishima M., Kaitna S., Glotzer M. (2002). Central spindle assembly and cytokinesis require a kinesin-like protein/RhoGAP complex with microtubule bundling activity. Dev. Cell.

[145] Tao L., Fasulo B., Warecki B., Sullivan W. (2016). Tum/RacGAP functions as a switch activating the Pav/kinesin-6 motor. Nat. Commun..

[146] Mishima M. (2016). Centralspindlin in rappaport’s cleavage signaling. Semin. Cell Dev. Biol..

[147] Fu C., Ward J.J., Loiodice I., Velve-Casquillas G., Nedelec F.J., Tran P.T. (2009). Phospho-regulated interaction between kinesin-6 KLP9p and microtubule bundler Ase1p promotes spindle elongation. Dev. Cell.

[148] Drechsler H., McAinsh A.D. (2016). Kinesin-12 motors cooperate to suppress microtubule catastrophes and drive the formation of parallel microtubule bundles. Proc. Natl. Acad. Sci. USA.

[149] McNally K.P., Panzica M.T., Kim T., Cortes D.B., McNally F.J. (2016). A novel chromosome segregation mechanism during female meiosis. Mol. Biol. Cell.

[150] Bieling P., Telley I.A., Surrey T. (2010). A minimal midzone protein module controls formation and length of antiparallel microtubule overlaps. Cell.

[151] Hu C.K., Coughlin M., Field C.M., Mitchison T.J. (2011). Kif4 regulates midzone length during cytokinesis. Curr. Biol..

[152] Nunes Bastos R., Gandhi S.R., Baron R.D., Gruneberg U., Nigg E.A., Barr F.A. (2013). Aurora b suppresses microtubule dynamics and limits central spindle size by locally activating Kif4a. J. Cell Biol..

[153] Zhu C., Zhao J., Bibikova M., Leverson J.D., Bossy-Wetzel E., Fan J.B., Abraham R.T., Jiang W. (2005). Functional analysis of human microtubule-based motor proteins, the kinesins and dyneins, in mitosis/cytokinesis using rna interference. Mol. Biol. Cell.

[154] Bieling P., Laan L., Schek H., Munteanu E.L., Sandblad L., Dogterom M., Brunner D., Surrey T. (2007). Reconstitution of a microtubule plus-end tracking system in vitro. Nature.

[155] Pereira A.L., Pereira A.J., Maia A.R., Drabek K., Sayas C.L., Hergert P.J., Lince-Faria M., Matos I., Duque C., Stepanova T. (2006). Mammalian clasp1 and clasp2 cooperate to ensure mitotic fidelity by regulating spindle and kinetochore function. Mol. Biol. Cell.

[156] Maton G., Edwards F., Lacroix B., Stefanutti M., Laband K., Lieury T., Kim T., Espeut J., Canman J.C., Dumont J. (2015). Kinetochore components are required for central spindle assembly. Nat. Cell Biol..

[157] Maiato H., Khodjakov A., Rieder C.L. (2005). *Drosophila* clasp is required for the incorporation of microtubule subunits into fluxing kinetochore fibres. Nat. Cell Biol..

[158] Chen Y., Hancock W.O. (2015). Kinesin-5 is a microtubule polymerase. Nat. Commun..

[159] Cole D.G., Chinn S.W., Wedaman K.P., Hall K., Vuong T., Scholey J.M. (1993). Novel heterotrimeric kinesin-related protein purified from sea urchin eggs. Nature.

[160] Craft J.M., Harris J.A., Hyman S., Kner P., Lechtreck K.F. (2015). Tubulin transport by IFT is upregulated during ciliary growth by a cilium-autonomous mechanism. J. Cell Biol..

[161] Henson J.H., Cole D.G., Terasaki M., Rashid D., Scholey J.M. (1995). Immunolocalization of the heterotrimeric kinesin-related protein KRP(85/95) in the mitotic apparatus of sea urchin embryos. Dev. Biol..

[162] Lecland N., Luders J. (2014). The dynamics of microtubule minus ends in the human mitotic spindle. Nat. Cell Biol..

[163] Goodwin S.S., Vale R.D. (2010). Patronin regulates the microtubule network by protecting microtubule minus ends. Cell.

[164] Hendershott M.C., Vale R.D. (2014). Regulation of microtubule minus-end dynamics by camsaps and patronin. Proc. Natl. Acad. Sci. USA.

[165] Jiang K., Hua S., Mohan R., Grigoriev I., Yau K.W., Liu Q., Katrukha E.A., Altelaar A.F., Heck A.J., Hoogenraad C.C. (2014). Microtubule minus-end stabilization by polymerization-driven camsap deposition. Dev. Cell.

[166] Rogers G.C., Rogers S.L., Schwimmer T.A., Ems-McClung S.C., Walczak C.E., Vale R.D., Scholey J.M., Sharp D.J. (2004). Two mitotic kinesins cooperate to drive sister chromatid separation during anaphase. Nature.

[167] Carmena M., Wheelock M., Funabiki H., Earnshaw W.C. (2012). The chromosomal passenger complex (CPC): From easy rider to the godfather of mitosis. Nat. Rev. Mol. Cell Biol..

[168] Pavin N., Tolic-Norrelykke I.M. (2013). Dynein, microtubule and cargo: A menage a trois. Biochem. Soc. Trans..

[169] Schmidt H., Carter A.P. (2016). Review: Structure and mechanism of the dynein motor ATPase. Biopolymers.

[170] Ananthanarayanan V., Schattat M., Vogel S.K., Krull A., Pavin N., Tolic-Norrelykke I.M. (2013). Dynein motion switches from diffusive to directed upon cortical anchoring. Cell.

[171] Laan L., Pavin N., Husson J., Romet-Lemonne G., van Duijn M., Lopez M.P., Vale R.D., Julicher F., Reck-Peterson S.L., Dogterom M. (2012). Cortical dynein controls microtubule dynamics to generate pulling forces that position microtubule asters. Cell.

[172] Gonczy P., Pichler S., Kirkham M., Hyman A.A. (1999). Cytoplasmic dynein is required for distinct aspects of mtoc positioning, including centrosome separation, in the one cell stage *Caenorhabditis elegans* embryo. J. Cell Biol..

[173] Vaisberg E.A., Koonce M.P., McIntosh J.R. (1993). Cytoplasmic dynein plays a role in mammalian mitotic spindle formation. J. Cell Biol..

[174] Gerdes K., Howard M., Szardenings F. (2010). Pushing and pulling in prokaryotic DNA segregation. Cell.

[175] Bharat T.A., Murshudov G.N., Sachse C., Lowe J. (2015). Structures of actin-like ParM filaments show architecture of plasmid-segregating spindles. Nature.

[176] Garner E.C., Campbell C.S., Weibel D.B., Mullins R.D. (2007). Reconstitution of DNA segregation driven by assembly of a prokaryotic actin homolog. Science.

[177] Bastos R.N., Cundell M.J., Barr F.A. (2014). Kif4a and pp2a-b56 form a spatially restricted feedback loop opposing aurora b at the anaphase central spindle. J. Cell Biol..

[178] Musacchio A. (2011). Spindle assembly checkpoint: The third decade. Philos. Trans. R. Soc. Lond. B Biol. Sci..

[179] Sullivan M., Morgan D.O. (2007). Finishing mitosis, one step at a time. Nat. Rev. Mol. Cell Biol..

[180] Parry D.H., O’Farrell P.H. (2001). The schedule of destruction of three mitotic cyclins can dictate the timing of events during exit from mitosis. Curr. Biol..

[181] Yuan K., O’Farrell P.H. (2015). Cyclin b3 is a mitotic cyclin that promotes the metaphase-anaphase transition. Curr. Biol..

[182] Afonso O., Matos I., Pereira A.J., Aguiar P., Lampson M.A., Maiato H. (2014). Feedback control of chromosome separation by a midzone Aurora B gradient. Science.

[183] Drechsler H., McAinsh A.D. (2012). Exotic mitotic mechanisms. Open Biol..

[184] Sullivan D.S., Huffaker T.C. (1992). Astral microtubules are not required for anaphase B in *Saccharomyces cerevisiae*. J. Cell Biol..

[185] Yeh E., Yang C., Chin E., Maddox P., Salmon E.D., Lew D.J., Bloom K. (2000). Dynamic positioning of mitotic spindles in yeast: Role of microtubule motors and cortical determinants. Mol. Biol. Cell.

[186] Bannigan A., Lizotte-Waniewski M., Riley M., Baskin T.I. (2008). Emerging molecular mechanisms that power and regulate the anastral mitotic spindle of flowering plants. Cell. Motil. Cytoskelet..

[187] Hayashi T., Sano T., Kutsuna N., Kumagai-Sano F., Hasezawa S. (2007). Contribution of anaphase B to chromosome separation in higher plant cells estimated by image processing. Plant Cell Physiol..

[188] Lee Y.R., Qiu W., Liu B. (2015). Kinesin motors in plants: From subcellular dynamics to motility regulation. Curr. Opin. Plant Biol..

[189] Struk S., Dhonukshe P. (2014). Maps: Cellular navigators for microtubule array orientations in arabidopsis. Plant Cell Rep..

[190] Miki T., Naito H., Nishina M., Goshima G. (2014). Endogenous localizome identifies 43 mitotic kinesins in a plant cell. Proc. Natl. Acad. Sci. USA.

[191] Cowan C.R., Hyman A.A. (2004). Asymmetric cell division in *C. elegans*: Cortical polarity and spindle positioning. Annu. Rev. Cell Dev. Biol..

[192] Hara Y., Kimura A. (2009). Cell-size-dependent spindle elongation in the *Caenorhabditis elegans* early embryo. Curr. Biol..

[193] Nguyen-Ngoc T., Afshar K., Gonczy P. (2007). Coupling of cortical dynein and G alpha proteins mediates spindle positioning in *Caenorhabditis elegans*. Nat. Cell Biol..

[194] Hayward D., Metz J., Pellacani C., Wakefield J.G. (2014). Synergy between multiple microtubule-generating pathways confers robustness to centrosome-driven mitotic spindle formation. Dev. Cell.

[195] Brugues J., Nuzzo V., Mazur E., Needleman D.J. (2012). Nucleation and transport organize microtubules in metaphase spindles. Cell.

[196] Walczak C.E., Vernos I., Mitchison T.J., Karsenti E., Heald R. (1998). A model for the proposed roles of different microtubule-based motor proteins in establishing spindle bipolarity. Curr. Biol..

[197] Murray A.W., Desai A.B., Salmon E.D. (1996). Real time observation of anaphase in vitro. Proc. Natl. Acad. Sci. USA.

[198] Civelekoglu-Scholey G., Cimini D. (2014). Modelling chromosome dynamics in mitosis: A historical perspective on models of metaphase and anaphase in eukaryotic cells. Interface Focus.

[199] Brust-Mascher I., Civelekoglu-Scholey G., Scholey J.M. (2015). Mechanism for anaphase B: Evaluation of “slide-and-cluster” versus “slide-and-flux-or-elongate” models. Biophys. J..

[200] Loughlin R., Heald R., Nedelec F. (2010). A computational model predicts *Xenopus* meiotic spindle organization. J. Cell Biol..

[201] Uehara R., Tsukada Y., Kamasaki T., Poser I., Yoda K., Gerlich D.W., Goshima G. (2013). Aurora B and Kif2a control microtubule length for assembly of a functional central spindle during anaphase. J. Cell Biol..

[202] Kellogg E.H., Howes S., Ti S.C., Ramirez-Aportela E., Kapoor T.M., Chacon P., Nogales E. (2016). Near-atomic cryo-EM structure of PRC1 bound to the microtubule. Proc. Natl. Acad. Sci. USA.

[203] Chen I.A. (2009). Cell division: Breaking up is easy to do. Curr. Biol..

